# Computational study of metal doped coronene quantum dots for formaldehyde sensing and adsorption in medical and environmental applications

**DOI:** 10.1038/s41598-025-32667-7

**Published:** 2025-12-13

**Authors:** Khaled Almansour, Hashem O. Alsaab, Mahboubeh Pishnamazi

**Affiliations:** 1 Department of Pharmaceutics, College of Pharmacy, University of Hail, Hail, Saudi Arabia; 2 Department of pharmaceutics and pharmaceutical technology, college of pharmacy, Taif University, Taif 21944, Saudi Arabia , Taif University, Taif, 21944 Saudi Arabia; 3 Institute of Research and Development, Duy Tan University, Da Nang, Vietnam; 4 School of Medicine and Pharmacy, Duy Tan University, Da Nang, Vietnam

**Keywords:** Formaldehyde, Graphene quantum dots, DFT/QTAIM calculation, Coronene, Adsorption, Chemistry, Environmental sciences, Materials science, Nanoscience and technology

## Abstract

In this study, the ability of pure and aluminum- and zinc-doped coronene as dual-purpose adsorbent/sensor platforms for formaldehyde (FA) detection in environmental and biomedical applications was computationally studied. All molecular structures were optimized individually and in combination with FA using density functional theory (DFT) at the B97D/6-311 + G(d) level of theory in the gas and water phases, and validated using WB97XD calculations. Analyses included geometric optimization, coherence energy, IR and UV spectra (TD-DFT), MEP mapping, HOMO-LUMO distributions, DOS plots, reactivity descriptors, dipole moment and polarizability, adsorption energy, recovery time, and electrical conductivity. Intermolecular interactions were analyzed using the theory of atoms in molecules (QTAIM) and non-covalent interaction (NCI) analyses. Among all structures, Al.Coronene@FA exhibited the strongest sensing ability with the highest adsorption energy of − 39.57 kcal/mol (water phase) and − 44.43 kcal/mol (gas phase), along with an extremely long recovery time of 1.08 × 10^13^ s (water) and 3.93 × 10^16^ s (gas). This was paired with the highest electrical conductivity of 2.85 × 10^9^ A m^− 2^ (water), confirming a strong charge-transfer mechanism. Conversely, Zn.Coronene@FA showed moderate adsorption (− 6.16 kcal/mol) and fast recovery time (3.34 × 10^− 12^ s), making it favorable for reusable sensing platforms. Optical studies revealed pronounced redshifts in λmax upon FA adsorption, particularly for Al.Coronene@FA with λmax = 579 nm and 694 nm (water phase), indicating excellent colorimetric detection capability. Overall, the combination of strong adsorption ability, high electrical conductivity, significant spectral shifts, and charge transfer indicates Al-doped coronene is a highly promising material for formaldehyde sensing, whereas Zn-doped coronene is better suited for rapid-response reusable sensors. These computational results establish a reliable foundation for developing coronene-based sensing and adsorption platforms for environmental and biomedical applications.

**Supplementary Information:**

The online version contains supplementary material available at 10.1038/s41598-025-32667-7.

## Introduction

Formaldehyde (FA) is a very reactive aldehyde that is common in both natural and anthropogenic environments. It is released from industrial production, fuel combustion, building materials, and consumer goods, making it one of the most common volatile organic compounds. Due to its toxicity, mutagenicity, and potential for causing cancer, formaldehyde is a significant concern for environmental health^1,2^. Qu et al. highlighted its harmful role as an environmental toxicant and that chronic exposure leads to oxidative stress, respiratory symptoms, and systemic toxicity, which potentially requires monitoring and regulation^3^. In addition to the environmental toxicity of formaldehyde, it has gained increased attention and interest in biomedical science as a potential biomarker of neurodegenerative disease, particularly Alzheimer’s disease (AD). A systematic review and meta-analysis by Chen et al. provided compelling evidence of significantly higher urinary formaldehyde among people with AD compared to controls, thus elevating the possibility of using non-invasive assessment of formaldehyde metabolites as a diagnostic tool^4^.

Given its dual role (as a toxic pollutant in the environment and as an essential clinical biomarker), formaldehyde is a molecule of exceptional interdisciplinary relevance. The development of formaldehyde monitoring and quantification will not only further environmental protection efforts but also provide new avenues for identifying, preventing, and treating Alzheimer’s disease. Standard detection methods for formaldehyde include spectrophotometry, chromatography, mass spectrometry, and electrochemical sensing. Although high sensitivity and accuracy can be achieved with these techniques, they face practical barriers. Many of these approaches require sophisticated instruments that may not always be available in routine laboratories, trained staff to operate these systems, and considerable capital and ongoing operating costs. These limitations mark an appropriate use of these standard methods impossible or impractical in many settings, especially resource-limited settings^5,6^. Despite these limitations, the increasing environmental and biomedical relevance of formaldehyde elucidates the need for efficient, low-cost, and simplified detection methods that yield reliable results in real time.

To address these issues, the development of nanomaterials with dual functions as adsorbents and sensors has garnered some interest. Carbon-based nanomaterials, particularly graphene quantum dots (GQDs), including coronene, corannulene, and ovalene, possess excellent physicochemical properties^7,8^.

Carbon-based nanomaterials, particularly graphene quantum dots (GQDs), have attracted significant attention due to their remarkable physicochemical properties, making them highly versatile for applications in electronics, energy storage, sensors, and biomedicine. GQDs are essentially small fragments of graphene with quantum-sized dimensions that exhibit unique electronic, optical, and chemical behaviors. Within this category, compounds such as coronene, corannulene, and ovalene stand out for their exceptional properties. Among these, coronene is widely regarded as the most popular and studied due to its superior properties. The superiority of coronene stems from its structural configuration and electronic properties. Its planar and highly aromatic structure enables efficient charge transport, and its broad composition enhances its photoluminescent properties. In addition, coronene’s chemical stability under various conditions, such as high temperatures or aggressive chemical environments, contributes to its desirability in practical applications. Compared with other graphene quantum dots such as coronene and ovalene, the larger size and better-defined structure of coronene offer more predictable and efficient performance in many technological applications, establishing its position as the most prominent member of the carbon-based nanomaterial family. In addition, coronene’s good electrical conductivity, mechanical strength, and chemical stability enhance the performance of this structure for interaction with target analytes and their detection, thus making it suitable for sensing or adsorption applications. Also, the unstable π electrons and tunability of surface functional groups provide strong adsorption capabilities and sensitive transmission of chemical signals. Properties such as these make coronene a promising candidate for designing efficient platforms for contaminant removal across various methods and rapid sensing applications^9,10^.

One topic of interest in recent papers is the versatile properties of coronenes as sensors and adsorbents. For example, Jadoon et al. used silver cluster (Ag6) decorated coronene as a non-enzymatic sensor for glucose and H2O2 detection. They highlighted its utility as a diagnostic tool for biomedical applications^11^. Kumar et al. studied the adsorption of adenine on pure and B/N/O/P doped coronene as a biosensor substrate for DNA detection and highlighted its utility in the medical field^12^. Ahmed et al. studied the use of Circum-doped coronene for catalysis, sensing, and energy storage applications, with a focus on environmental monitoring, where they used this material as an N2O gas sensor and reported the advantages of heteroatom doping in improving the electronic and sensing properties^13^.

Considering that formaldehyde has relevance as an environmental contaminant and a biomarker for Alzheimer’s disease, this investigation will explore its binding onto pristine coronene and aluminum- and zinc-doped coronene to study the role of doping with aluminum and zinc means to have them react chemically more energetically and to alter the electronic system of the coronene structure to design active sites to enhance the binding reaction energies and overall capacity with formaldehyde molecules. Aluminum, with a polarized cationic state resulting from electron removal, can act as a Lewis acid, forming stronger interactions with electron-rich molecules such as formaldehyde^14^. In addition, zinc, with its partially filled d orbitals, can facilitate charge transfer and improve the binding capacity and sensitivity of coronene to formaldehyde and other organics^15^. As a larger objective, we plan to design a dual-purpose platform capable of binding and detecting formaldehyde to support environmental protection and public health. To achieve this goal, we will investigate the binding and energy interactions of the formaldehyde and coronene (in the gas/water-phase) components using density functional theory (DFT) and time-dependent DFT (TD-DFT) based modelling along with quantum theory of atoms in molecules (QTAIM) and then predict how they interact without practical experimentation (i.e., avoiding the hurdles of drawbacks with lab experiments) and safety concern in experiments^16–18^. We expect that the results of this research will enable efficient graphene-based platforms (specifically coronene) to monitor and remove formaldehyde, which may be applicable in environmental and biomedical sectors.

## Computational details

**Fig. 1:**
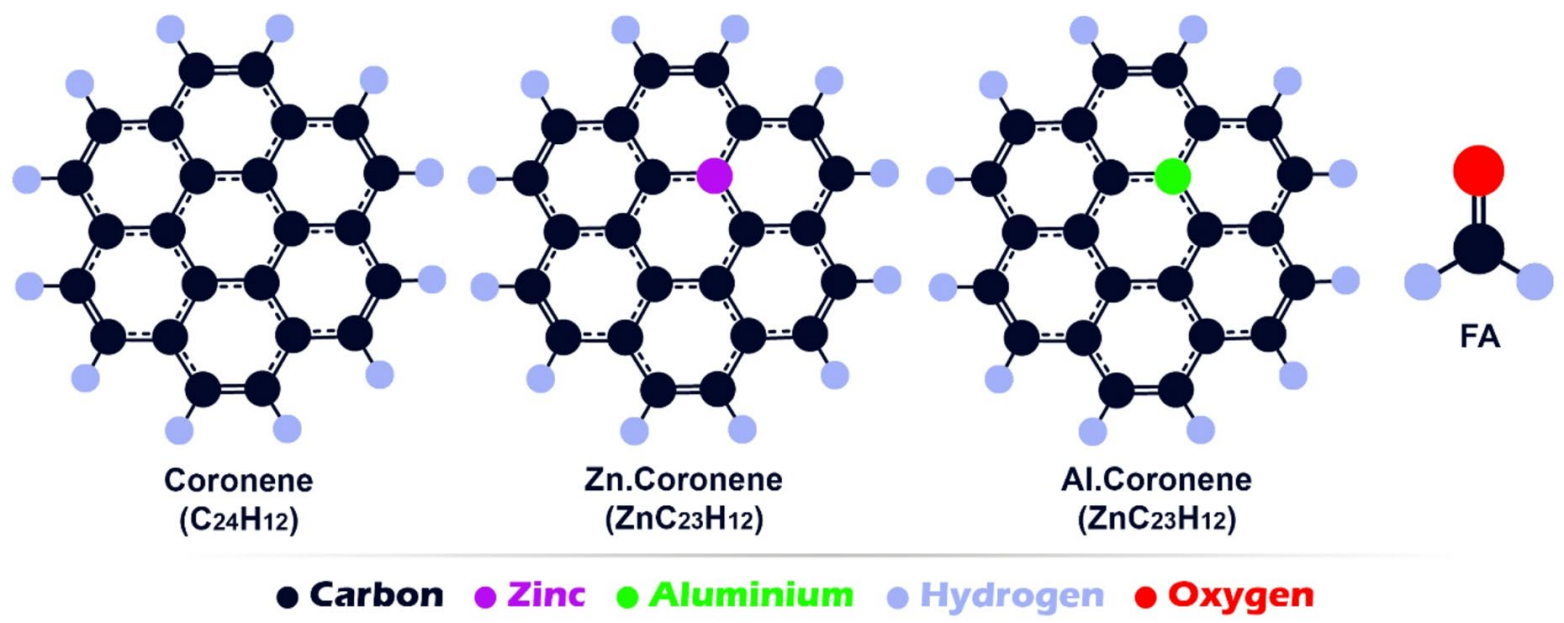
Structures simulated in this work.

First, all molecular structures (Coronene (C_24_H_12_), Al-doped Coronene (Al.Coronene (AlC_23_H_12_)), Zn-doped Coronene (Zn.Coronene (ZnC_23_H_12_)), formaldehyde (FA), and their complexes) were designed using GaussView 6.0. In the next step, the geometric structures of each molecule and its complex were optimized at the DFT/B97d/6-311 + G(d) level in the water phase (using the Conductor-like Polarizable Continuum Model (CPCM)) and gas phase with Gaussian 09 W software (see Fig. 1) (The X, Y, Z coordinates of each structure were reported in the supplementary data for the purpose of future calculation repeatability (Table S1))^19–21^. In order to confirm the complete geometric optimization of each of the designed structures, geometric optimization convergence plots for each of them were reported in the supplementary data (see Figs. S1, S2, and S3 in the Supplementary Data). The B97D functional was chosen because it includes empirical dispersion corrections, allowing accurate modeling of weak interactions such as van der Waals forces, which are important in adsorption studies^22^. The water phase was considered to mimic realistic environmental or biological conditions, as formaldehyde often interacts with surfaces in aqueous media, ensuring that the predictions are more relevant to practical applications^23^. It is necessary to explain that while the CPCM approach is appropriate for modelling solvent effects, it represents the medium as a dielectric continuum. Therefore, it does not explicitly account for individual solvent molecules that could compete with or facilitate FA adsorption (especially around polar metal sites). Including explicit solvent molecules in future models may provide a more accurate description of these local interactions. Also, the gas phase is chosen to study the interaction between coronene and formaldehyde because it closely simulates real atmospheric conditions, where both compounds exist in the gas phase. This enables more accurate analysis of their interaction, free from the influence of solvents or other environmental factors.

To ensure the reliability and accuracy of the chosen computational method (B97D/6-311 + G(d)), all calculations were repeated using the WB97XD functional with the same basis set (6-311 + G(d)) and in aqueous phase using the Conductor-like Polarizable Continuum Model (CPCM) to simulate the solvent effect. This step was done to evaluate how well the results obtained with the initial method (B97D/6-311 + G(d)) matched those obtained with a different functional (WB97XD), particularly in the presence of water as the solvent. The WB97XD functional was selected due to its incorporation of empirical dispersion corrections and long-range exact exchange, which enable accurate treatment of noncovalent interactions such as π–π stacking and hydrogen bonding. This functional offers a suitable balance between computational efficiency and reliability, rendering it well-suited for systems where dispersion and long-range effects play a significant role^24^. Comparing the trends in the results obtained from both methods can further confirm the accuracy of the calculations.

Frequency calculations were performed at the same theoretical level and no imaginary frequencies were observed. The absence of imaginary frequencies indicates that all the studied structures are stable and not in a transition state.

Also, the optical properties (UV spectrum and exciton energy) of each structure in the presence and absence of formaldehyde were calculated using TD-DFT theory at the same theoretical level.

The cohesive energy (E_Coh_) was calculated using Eq. 1 to evaluate the effect of doping on the stability of graphene.

1 $$\:{E}_{Coh}=\frac{{\sum\:}_{i}{E}_{i}^{atom}-{E}_{total}^{solid}}{n}$$

Where: $\:{E}_{i}^{atom}$ = energy of the isolated atom (Such as: C, H, Al, and Zn), $\:{E}_{total}^{solid}$= The total energy of Coronene or its doping forms, and n = total number of atoms^25^. The term “energy of the isolated atom” indeed refers to the ground-state energy of each atom (C, H, Al, Zn) calculated individually at the same computational level as the complexes (B97D/6-311+G(d) in the water phases using CPCM). For each atom, we performed a spin-polarized calculation using the correct ground-state multiplicity (e.g., triplet for C, doublet for O), and these values were directly inserted into Eq. 1 to ensure methodological consistency and reproducibility.

Reactivity parameters such as energy gap (HLG), chemical hardness ($\:{\upeta\:}$), chemical softness (S), and chemical potential ($\:{\upmu\:}$) were calculated using Eqs. 2–5, respectively. In these equations, E_HOMO_ represents the energy of HOMO frontier orbitals and E_LUMO_ represents the energy of LUMO frontier orbitals. All values are in terms of eV^26,27^.

2 $$\:HLG=\left|{E}_{HOMO}-{E}_{LUMO}\right|$$

3 $$\:{\upeta\:}=\raisebox{1ex}{$(-{\mathrm{E}}_{\mathrm{H}\mathrm{O}\mathrm{M}\mathrm{O}}-(-{\mathrm{E}}_{\mathrm{L}\mathrm{U}\mathrm{M}\mathrm{O}}\:\left)\right)$}\!\left/\:\!\raisebox{-1ex}{$2$}\right.$$

4 $$\:S=1/2{\upeta\:}$$

5 $$\:{\upmu\:}=-(-{\mathrm{E}}_{\mathrm{H}\mathrm{O}\mathrm{M}\mathrm{O}}+(-{\mathrm{E}}_{\mathrm{L}\mathrm{U}\mathrm{M}\mathrm{O}}\left)\right)/2$$

The maximum charge transferred (ΔNmax) and charge transfer based on electrophilicity (ECT) were evaluated using Eqs. 6 and 7, respectively. In Eq. 7, α represents the charge transferred by the complex and β represents the maximum charge transferred by the sensor. A positive value of ECT indicates that the sensor behaves as an electron donor and vice versa^28^.

6 $$\:{\varDelta\:N}_{max}=-\raisebox{1ex}{$\mu\:$}\!\left/\:\!\raisebox{-1ex}{$\eta\:$}\right.$$

7 $$\:ECT={\left({{\Delta\:}N}_{max}\right)}_{\alpha\:}-{\left({{\Delta\:}N}_{max}\right)}_{\beta\:}$$

Recovery time (τ) and electrical conductivity ($\:{\upsigma\:}$) were calculated using Eqs. 8 and 9, respectively.

8 $$\:\tau\:={V}_{0}^{-1}\times\:\mathrm{e}\mathrm{x}\mathrm{p}(-\frac{{E}_{ads}}{{k}_{B}T})$$

9 $$\:{\upsigma\:}=A{T}^{3/2}{e}^{(-Eg/2KT)}$$

In these equations: A = Richardson constant (6 × 10^5^ A.m^− 2^), k_B_= Boltzmann constant, E_ads_= adsorption energy, V_0_ = attempt frequency (10^12^ s^− 1^), and, T = temperature (298 K)^29,30^.

The adsorption energies (Eads) for each complex were calculated using Eq. 10.

10 $$\:{E}_{ads}={E}_{\left(R\right)-\mathrm{C}\mathrm{o}\mathrm{r}\mathrm{o}\mathrm{n}\mathrm{e}\mathrm{n}\mathrm{e}@FA}-\left({E}_{FA}+{E}_{\left(R\right)-\mathrm{C}\mathrm{o}\mathrm{r}\mathrm{o}\mathrm{n}\mathrm{e}\mathrm{n}\mathrm{e}}\right)+{E}_{BSSE}$$

In these equations: E_(R)−Coronene@FA_= Total energy of the optimized complex (FA adsorbed on R-functionalized Coronene, where R = Al, Zn, or pure Coronene), E_FA_ = Energy of formaldehyde (optimized geometry), E_(R−)Coronene_ refers to the energy of doped Coronene in which one carbon atom is replaced by Zn or Al, and E_BSSE_= Basis Set Superposition Error correction^31^. The E_BSSE_ term was corrected using the Counterpoise (And by adding the expression counterpoise = 2 in the calculation method) method based on the Boys–Bernardi formalism, ensuring accurate adsorption energies for all complexes.

Equations 11 and 12 were used to investigate the dipole moment ($\:\mu\:$) and polarizability ($\:\alpha\:$)^32^.

11 $$\:\mu\:=\sqrt{{\mu\:}_{x}^{2}+{\mu\:}_{y}^{2}+{\mu\:}_{z}^{2}}$$

12 $$\:\alpha\:=\frac{1}{3}\left({\alpha\:}_{xx}+{\alpha\:}_{yy}+{\alpha\:}_{zz}\right)$$

These computational analyses offer valuable insights into the (R)-Coronene@FA interactions, providing a solid foundation for the rational design of graphene-based sensors and adsorbents.

## Results and discussion

### Bond length/angle

**Fig. 2:**
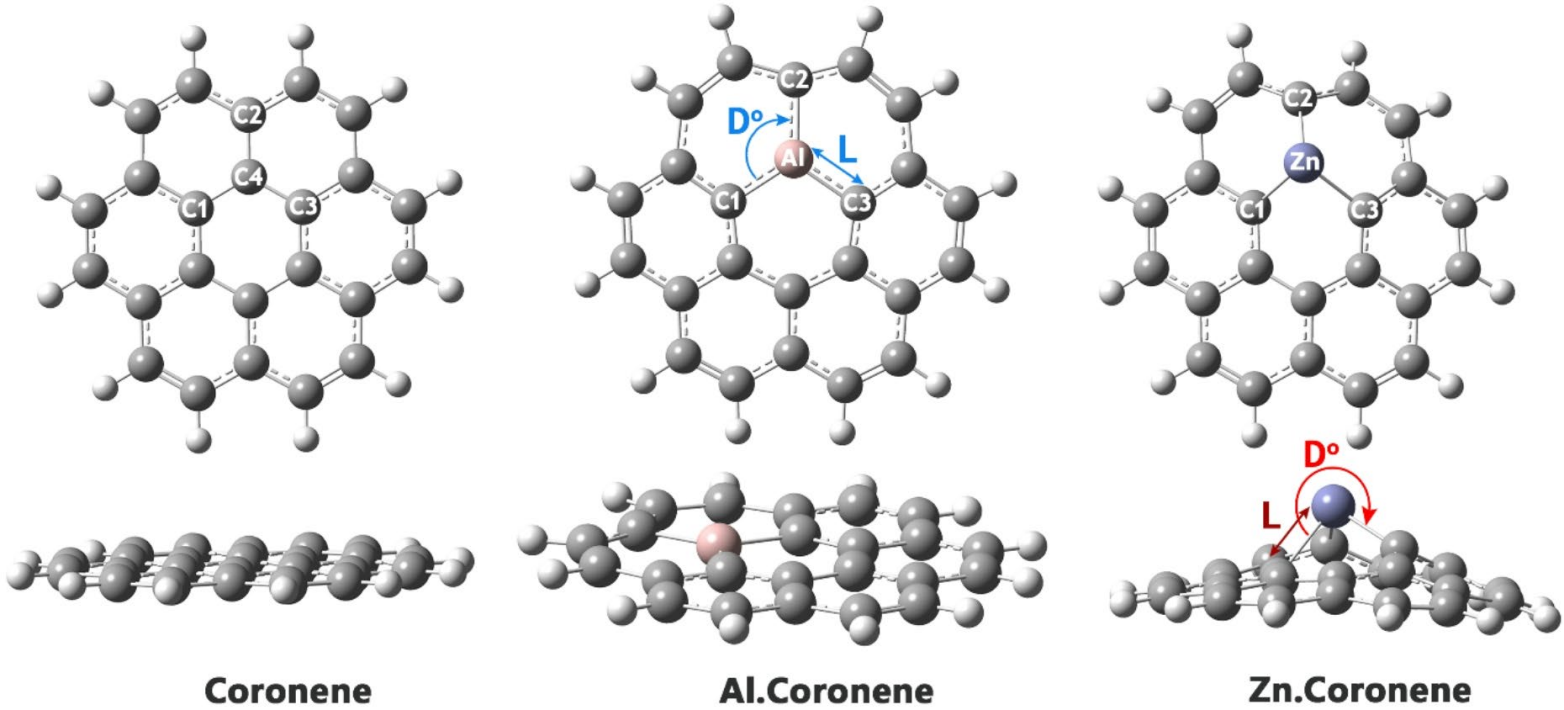
Optimized structure of each of the designed structures in the absence of analyte FA.

Examining bond lengths (L) and angles (D) is crucial when doping a material because the introduced dopant atoms disrupt the local atomic structure, causing bonds to stretch, compress, or angles to bend. These subtle atomic-scale distortions directly dictate the material’s macroscopic electronic, conductive, and mechanical properties, making their measurement essential for understanding and predicting how doping will ultimately alter the material’s performance^33^. In this regard, the bond lengths and angles between necessary atoms (see Fig. 2) in each of the designed structures were computationally studied, and the results are reported in Table 1.

**Table 1:** Bond length/angle between some important atoms in each of the structures studied in this work.

Structure	L (Å)		D (°)	
B97D (water solvent)				
Coronene	C1-C4	1.430	C1-C4-C3	119.9
	C2-C4	1.430	C1-C4-C2	120.0
	C3-C4	1.430	C2-C4-C3	120.0
Al.Coronene	C1-Al	1.799	C1-Al-C3	112.68
	C2-Al	1.819	C1-Al-C2	123.65
	C3-Al	1.799	C2-Al-C3	123.65
Zn.Coronene	C1-Zn	2.17	C1-Zn-C3	79.42
	C2-Zn	1.99	C1-Zn-C2	82.86
	C3-Zn	1.98	C2-Zn-C3	101.23
B97D (gas phase)				
Coronene	C1-C4	1.430	C1-C4-C3	120.00
	C2-C4	1.429	C1-C4-C2	119.99
	C3-C4	1.430	C2-C4-C3	119.99
Al.Coronene	C1-Al	1.798	C1-Al-C3	112.71
	C2-Al	1.819	C1-Al-C2	123.64
	C3-Al	1.798	C2-Al-C3	123.64
Zn.Coronene	C1-Zn	2.05	C1-Zn-C3	86.40
	C2-Zn	1.95	C1-Zn-C2	88.98
	C3-Zn	1.93	C2-Zn-C3	108.41
WB97XD (water solvent)				
Coronene	C1-C4	1.42	C1-C4-C3	120
	C2-C4	1.40	C1-C4-C2	119.99
	C3-C4	1.42	C2-C4-C3	119.99
Al.Coronene	C1-Al	1.78	C1-Al-C3	112.66
	C2-Al	1.80	C1-Al-C2	123.66
	C3-Al	1.78	C2-Al-C3	123.66
Zn.Coronene	C1-Zn	2.30	C1-Zn-C3	86.79
	C2-Zn	2.29	C1-Zn-C2	39.25
	C3-Zn	1.99	C2-Zn-C3	83.94

In the water phase, the bond lengths for Coronene (C1-C4, C2-C4, and C3-C4) are reported as 1.430 Å, suggesting consistent carbon-carbon bond lengths within the molecule. The bond angles between these atoms (C1-C4-C3, C1-C4-C2, and C2-C4-C3) are approximately 120°, which indicates that the molecule maintains a nearly ideal sp2 hybridization geometry, typical for aromatic systems. This is expected as coronene is a polycyclic aromatic hydrocarbon. For the Al.Coronene complex, the Al-C1 bond is 1.799 Å, and the Al-C2 bond is slightly longer at 1.819 Å, reflecting the typical bond length variation seen in metal-organic complexes. The bond angles (C1-Al-C3, C1-Al-C2, and C2-Al-C3) are in the range of 112.68° to 123.65°, suggesting some deviation from ideal tetrahedral geometry, which is common for coordination complexes. In the Zn.Coronene complex, the Zn-C1 bond is 2.17 Å, and the Zn-C2 and Zn-C3 bonds are slightly shorter at 1.99 Å and 1.98 Å, respectively. The bond angles (C1-Zn-C3, C1-Zn-C2, and C2-Zn-C3) show some significant variation, with angles ranging from 79.42° to 101.23°, which suggests a more distorted geometry typical of metal-ligand interactions involving larger metal centers like zinc.

It should be noted, Zn-doping in coronene leads to significant distortions in bond angles, with C-Zn-C angles ranging between 84° and 104°, deviating from the ideal 120° of pristine coronene. This distortion suggests non-planarity, likely due to the size and coordination requirements of the Zn atom. As a result, the doping introduces strain into the lattice, reducing the overall stability of the structure, as evidenced by a decrease in cohesive energy. Such geometric distortions may hinder the formation of stable Zn-doped Coronene, as strain can complicate the synthesis process and potentially affect the material’s long-term stability. This observation highlights the importance of considering the physical implications of metal doping on the formation of graphene-based nanomaterials. It suggests that further optimization of synthetic strategies is necessary to accommodate the strain introduced by Zn-doping.

In the gas phase, the bond lengths for Coronene (C1-C4, C2-C4, and C3-C4) remain essentially the same (1.430 Å, 1.429 Å, and 1.430 Å), indicating that the molecular structure in the gas phase is almost identical to that in water. The bond angles are also very close to 120°, maintaining the expected aromatic geometry. For the Al.Coronene complex in the gas phase, the Al-C bond lengths are almost identical to those in the water phase (1.798 Å for C1-Al and 1.819 Å for C2-Al), and the bond angles are also very similar, suggesting minimal influence of the phase change on the coordination geometry of the aluminum center. The Zn.Coronene complex in the gas phase shows slightly shorter Zn-C bonds (C1-Zn = 2.05 Å, C2-Zn = 1.95 Å, and C3-Zn = 1.93 Å) compared to the water phase. The bond angles are also different, with angles such as C1-Zn-C3 = 86.40° and C2-Zn-C3 = 108.41°, showing that the zinc coordination geometry is slightly altered in the absence of a solvent, likely due to the absence of solvent interactions that would otherwise stabilize the structure.

The comparison between the B97D and WB97XD methods reveals a high degree of overlap, suggesting that the results obtained from these two methods are consistent and reliable. For example, in both methods, the bond lengths in Coronene are nearly identical (1.430 Å for B97D and 1.42 Å for WB97XD), and the bond angles are also very similar (120° for C1-C4-C3 and C1-C4-C2 in both methods). Similarly, the bond lengths in Al.Coronene (Al-C1 = 1.799 Å for B97D and 1.78 Å for WB97XD) and Zn.Coronene (C1-Zn = 2.17 Å for B97D and 2.30 Å for WB97XD) exhibit good overlap, further confirming that the methods are producing consistent results for different metal-organic complexes. Thus, the close agreement between the B97D and WB97XD methods in both the water and gas phases provides strong validation for the computational approach used in this study, indicating that these methods are appropriate for simulating the geometries of the molecules and complexes under the conditions considered.

### Cohesive energy

**Fig. 3:**
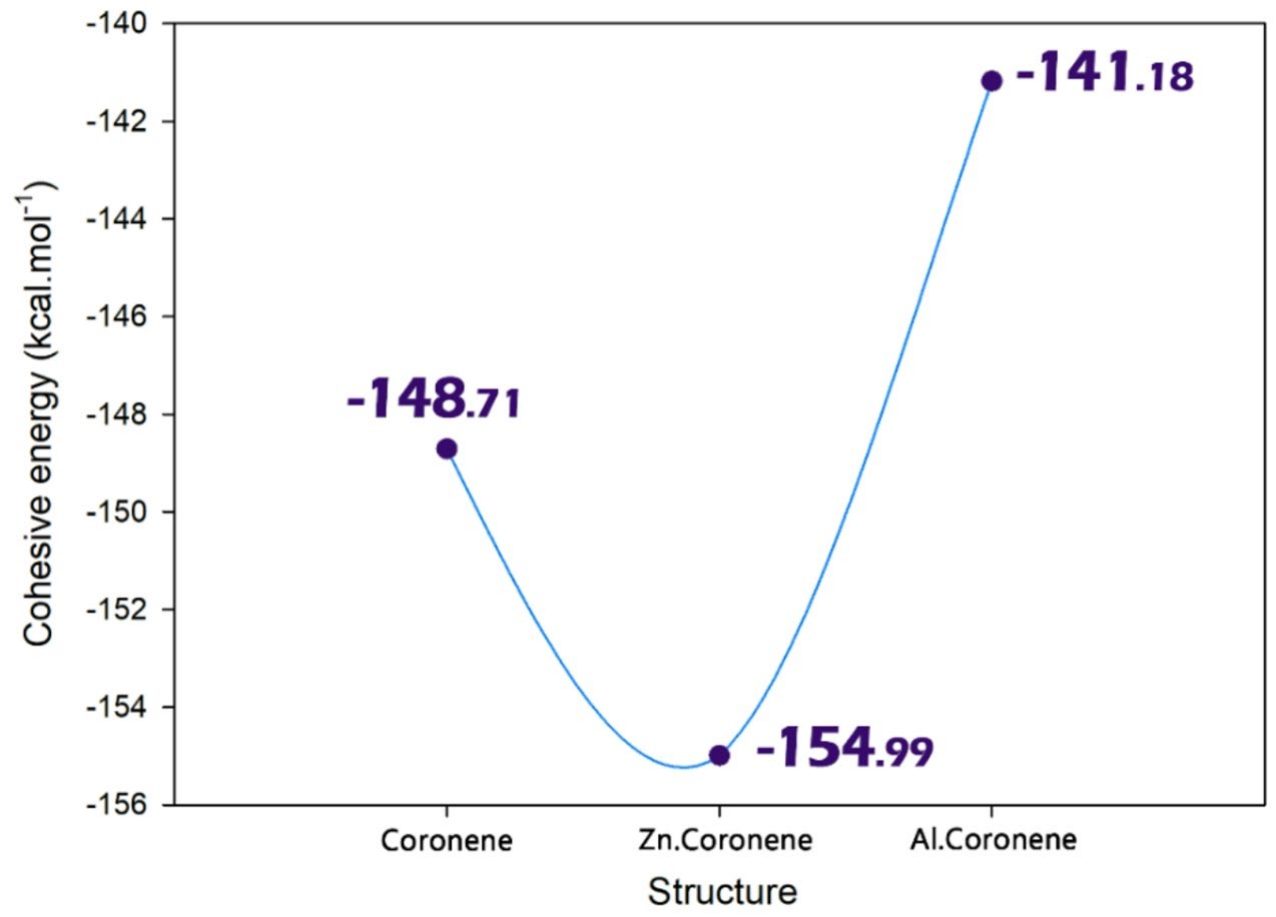
Effect of Al and Zn doping on the cohesive energy of coronene.

Cohesive energy is the energy that holds a material’s atoms together. In structural design, it is a critical measure of a material’s intrinsic stability and mechanical strength, determining its resistance to deformation or failure. When doping a structure, the introduction of foreign atoms disrupts the ideal host lattice, which typically lowers the cohesive energy by introducing strain and less optimal bonds^34^. Based on this explanation, the coherence energy was calculated for each of the designed structures and the results were reported in Fig. 3.

The cohesive energy of the three systems reveals a clear trend that directly corresponds to the structural distortions noted earlier from the bond length and angle analysis (Coronene > Al.Coronene > Zn.Coronene). The cohesive energy values reported in this work are expressed in kcal/mol, following the common convention where more negative values indicate greater structural stability. Our calculated value for pristine coronene (− 148.71 kcal/mol) is consistent with previous reports, where a similar magnitude of cohesive energy has been obtained using dispersion-corrected DFT calculations. For instance, Fedorov et al. reported a cohesive energy of approximately 1.48 eV per molecule (≈ − 34.3 kcal/mol), which corresponds to the interaction per isolated coronene molecule within the crystalline lattice. However, it is well established that multiple coronene molecules exist within each crystallographic unit cell, and when this multiplicity is considered, the total cohesive energy appropriately scales to values in the range of − 135 to − 150 kcal/mol. This places our calculated value of − 148.71 kcal/mol in excellent agreement with the literature and confirms that our results are fully consistent with experimental and dispersion-corrected DFT findings, provided that the unit-cell-based interpretation of cohesive energy is taken into account^35^. The cohesive energy of the aluminum (Al) doped coronene came in at − 141.18 kcal/mol indicating reduced stability overall within the structure which is supported by the previously noted elongated C-Al bonds and concurrent bond angle distortions (i.e., smaller bond angles) introducing strain and weakened cohesive binding forces. The most significant change in cohesive energy values was noted with zinc (Zn) doping with a value of − 145.99 kcal/mol indicating the respective drop in stability, which also corresponded exactly with the most structural deformation within the Zn.Coronene structure with the longest C–Zn bonds and most compressed bond angles indicating that a Zn dopant introduces the most instability within the lattice (These findings are consistent with the results obtained for bond length/angle).

### IR spectrum

**Fig. 4:**
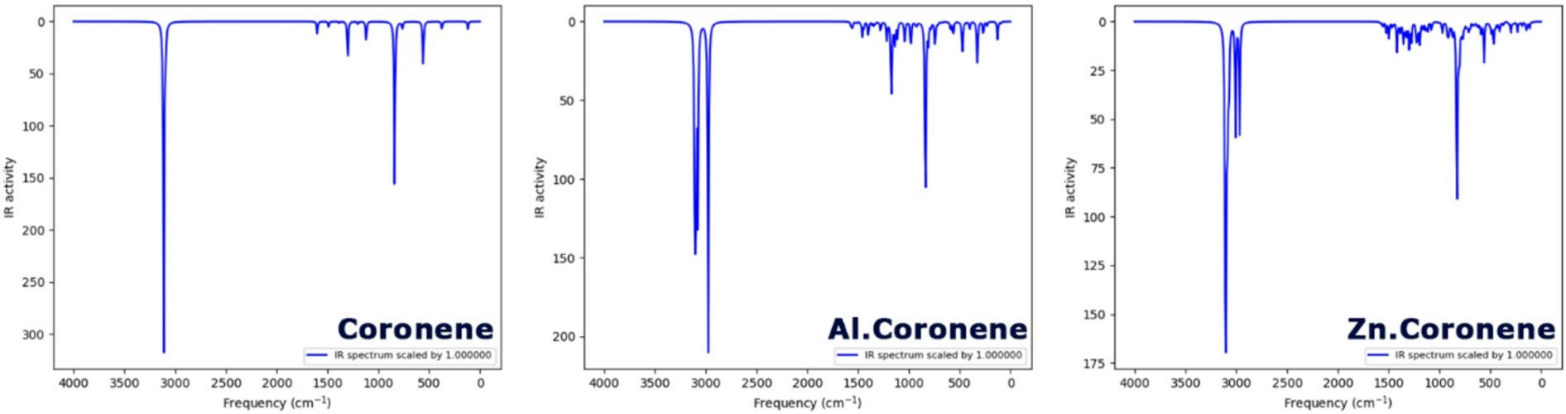
IR spectrum of each of the structures designed in this work.

Investigating the infrared (IR) spectrum is critical to verifying and validating designed structures. It provides experimental evidence or a fingerprint of the chemical bonds and functional groups present in the material. Each bond vibrates at a specific frequency, and by comparing the observed IR adsorption peaks properties (position, shape, and intensity around a characteristic peak) with the predicted adsorption properties of the theoretical model, one can substantiate the formation of a specific bond, identify the presence of an undesirable functional group, and/or recognize the presence of structural distortions^34^. The IR spectrum of each designed structure is depicted in Fig. 4.

The calculated IR spectrum of pristine Coronene reproduces the characteristic vibrational features of corannulene-based graphene fragments. A sharp and intense band is observed in the 3050–3100 cm^-1^ region, which corresponds to the aromatic C–H stretching vibrations of the peripheral hydrogens. Additional adsorptions appear around 1600 cm^-1^, attributed to C = C skeletal stretching of the aromatic network, while weaker bands in the 1200–1000 cm^-1^ region arise from in-plane C–H bending and C–C vibrations. The fingerprint region between 1000 and 700 cm^-1^ shows out-of-plane C–H bending modes typical for polycyclic aromatic hydrocarbons. These calculated features are in very good agreement with reported experimental IR spectra of corannulene and related PAHs, which consistently show strong aromatic C-H stretching near 3050 cm^-1^ and ring skeletal bands near 1600 cm^-1^^36^.

Also, the aromatic C-H stretching modes appear to remain, as seen near 3000 cm^-1^ but showing slight broadening and a red-shift with respect to some of the pristine structure indicating perturbation of the π-electron system by aluminum in the Al-doped system (Al.Coronene). In the low-frequency region below 600 cm^-1^ are several new bands, which may tentatively be assigned to Al-C and/or Al-O vibrations at the site of doping. The modes also show splitting around 1200 –1000 cm^-1^, which is again an indication of the presence of a local distortion across the lattice. Furthermore, in the far-IR region, changes in intensity and new metal-ligand vibrations were also noted, consistent with previous experimental studies of Al modified graphene composites^37^.

The Zn-doped system (Zn.Coronene) experiences similar changes as observed previously, with no loss of the aromatic stretching vibrations in the 3000 and 1600 cm^-1^ regions; although there is a different intensity, and it is broader in these two regions for Zn.Coronene. The most notable feature is the appearance of new strong adsorptions in the region of less than 500 cm^-1^, which have been shifted even lower than in the Al-doped system. This frequency decrease relates to the much heavier atomic weight of Zn than that of Al, which results in lower vibrational frequencies for the Zn-C and Zn-O bond modes. FT-IR studies of Zn-doped carbon dots and ZnO-GQD composites also observed very similar low-frequency bands in the region of 300–500 cm^-1^, which demonstrates that vibrations corresponding to Zn-C and Zn-O exist^38^.

### Molecular electrostatic potential (MEP) mapping and interaction prediction

Molecular Electrostatic Potential (MEP) analysis is a very effective approach for identifying the most likely interaction points between molecules, as it provides and predicts the regions of a molecule that are either electron-dense and therefore favorable for nucleophilic attack or electron-poor and therefore favorable sites for electrophilic attack, as well as regions favorable for non-covalent interactions like hydrogen bonding, halogen bonding, and electrostatic attraction or repulsion. The color contours of an MEP surface and its interpretations is very intuitive, with red regions being the most negative electrostatic potential where a positively charged species would be attracted to an electron-rich site of the molecule. Blue regions are the most positive potential, indicating regions that are electron-deficient and can be susceptible to attack from a nucleophile. Shades of green indicate neutral or nearly-zero electrostatic potential^39,40^.

**Fig. 5:**
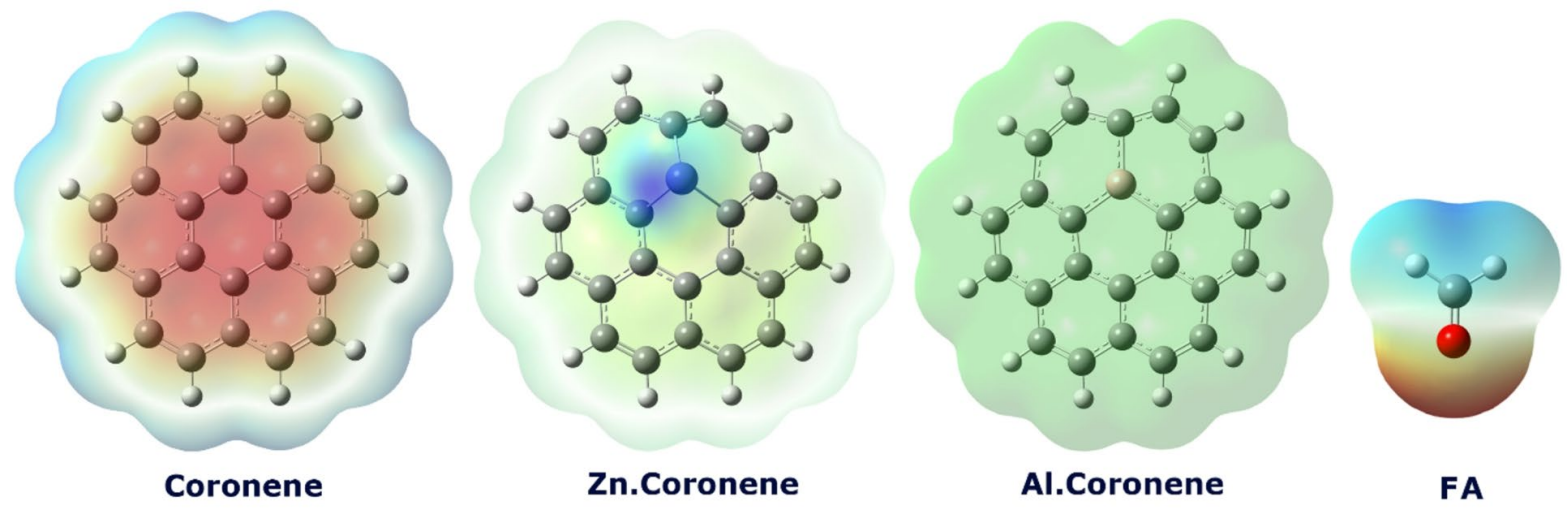
MEP contours for each of the structures studied in this work.

For the pristine Coronene, the red (electron-rich) center and blue (electron-deficient) edges create distinct regions. Formaldehyde, with its electron-rich red oxygen and electron-poor blue hydrogens, will most likely orient itself such that its hydrogen atoms (blue) interact electrostatically with the electron-rich central ring (red) of the coronene, suggesting a potential weak hydrogen-bond-like interaction.

In the Zn.Coronene, the presence of a strongly electron-deficient (blue) zinc atom dominates the electrostatic landscape. This creates a highly favorable binding site for the electron-rich oxygen atom (red) of formaldehyde. Consequently, the most stable complex is predicted to form via a dative or electrostatic interaction between the carbonyl oxygen of formaldehyde and the Zn atom.

For the Al.Coronene, the Al atom shows only a faint blue color, indicating a weakly positive character, while the carbon framework is largely neutral (green). This suggests a significantly weaker overall driving force for electrostatic interaction with formaldehyde compared to the other structures.

It is important to clarify that the MEP and ECT results are fully consistent and describe complementary aspects of the interaction mechanism. The red region around the oxygen atom of formaldehyde in the MEP map indicates a highly electron-rich site, suggesting that the carbonyl oxygen acts as the preferred nucleophilic center during adsorption. Accordingly, the ECT parameter shows negative values for all complexes, which confirms that FA behaves as the electron donor while the coronene-based surfaces function as electron acceptors. Thus, the electron density originates mainly from the lone pair on the oxygen of FA and is transferred to the doped coronene frameworks, consistent with a Lewis acid-base interaction.

**Fig. 6:**
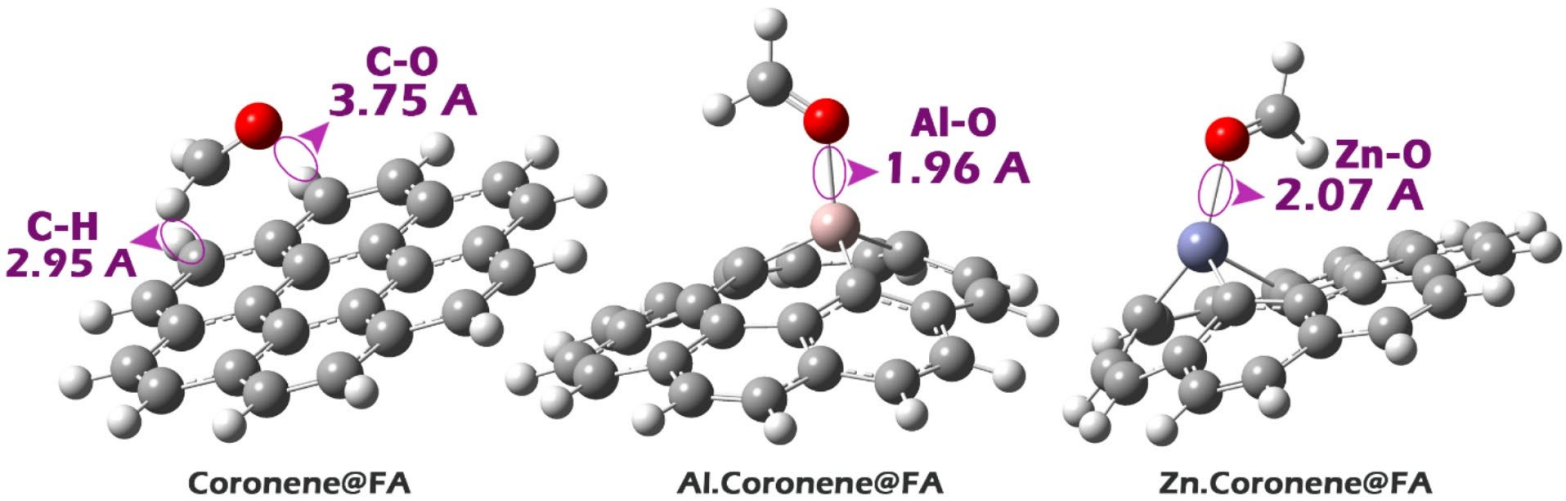
Optimized structure of each of the complexes designed in this study.

Any complex formation would likely be very weak and involve a non-specific interaction between the faintly blue Al site and the red oxygen of formaldehyde (see Fig. 5). Based on the results of the MEP analysis, each of the considered complexes was designed. The optimal shape of each of the studied complexes is shown in Fig. 6.

**Table 2:** Bond length changes for the Coronene, Zn.Coronene and Al.Coronene structures calculated using the two computational methods B97D and WB97XD, in both water and gas solvent phases.

Structure	Bond type	Bond length (Å)
B97D (water solvent)		
Coronene@FA	C-O	3.67
	C-H	2.93
Zn.Coronene@FA	Zn-O	2.13
Al.Coronene@FA	Al-O	1.95
B97D (gas)		
Coronene@FA	C-O	3.72
	C-H	2.93
Zn.Coronene@FA	Zn-O	2.09
Al.Coronene@FA	Al-O	1.92
WB97XD (water solvent)		
Coronene@FA	C-O	3.65
	C-H	3.00
Zn.Coronene@FA	Zn-O	1.98
Al.Coronene@FA	Al-O	1.93

Table 2 reports bond lengths for different molecular systems using two computational methods, B97D and WB97XD, in both the water solvent and gas phases. For Coronene@FA, in the water solvent, B97D predicts a C-O bond length of 3.67 Å, and WB97XD predicts 3.65 Å. These values are relatively long compared to typical C-O bonds, suggesting a weak or stretched bond. The C-H bond lengths in both methods are similarly reported, with B97D showing 2.93 Å and WB97XD showing a slightly longer bond at 3.00 Å, indicating a somewhat elongated C-H bond.

For the Zn.Coronene@FA complex, B97D in the water phase predicts a Zn-O bond length of 2.13 Å, while WB97XD predicts a shorter bond length of 1.98 Å. This indicates that WB97XD suggests a slightly stronger zinc-oxygen interaction compared to B97D. In the gas phase, B97D shows a slight decrease in the Zn-O bond length (2.09 Å), indicating a stronger zinc-oxygen interaction in the gas phase compared to the water solvent. For the Al.Coronene@FA complex, B97D reports an Al-O bond length of 1.95 Å in the water solvent, while WB97XD reports a slightly shorter bond length of 1.93 Å. In the gas phase, B97D reports a slight decrease in the Al-O bond length to 1.92 Å, which further confirms a stronger Al-O interaction in the gas phase.

The minimal difference between the B97D and WB97XD results indicates good agreement between the two methods. Overall, while there are small differences between the methods, the bond length values for the key interactions (C-O, C-H, Zn-O, and Al-O) are in close agreement. As a result, the interaction distances calculated from both the B97D and WB97XD methods follow the order Coronene@FA > Zn.Coronene@FA > Al.Coronene@FA, which not only indicates that doping, especially with Al and Zn, leads to a closer and potentially stronger association with the formaldehyde molecule, but also shows the same trend (qualitatively) in the obtained results. This overlap between the B97D and WB97XD methods, especially in the water phase, highlights that both methods produce consistent results and validate the computational approach used for these systems. The minimal variations between the methods further suggest that they provide reliable predictions of bond lengths for the molecular systems studied.

### Reactivity descriptors

The energy gap (HLG), chemical hardness (η), chemical softness (S), and chemical potential (µ) serve as indicators of reactivity and electronic stability for a sensing application. A small HLG and a large S, would indicate a high level of chemical reactivity with little kinetic stability and designates a material that is more reactive with the target analyte, which is ideal in situations requiring high sensitivity^41^. In contrast, a large HLG paired with a large η would indicate low reactivity and stable kinetics, which could be advantageous for sensor selectivity. Chemical potential will determine the direction of electron flow through sensing and could predict whether the material will be an electron donor or acceptor when sensing. ECT also indicates the direction of charge flow in a molecule^42^. Each of these properties are calculated in the presence of and absence of FA and the values are summarized in Table 3.

**Table 3:** Reactivity parameters calculated using the B97D (Water Solvent), B97D (Gas Phase) and WB97XD (Water Solvent) methods (9 with the same basis set (6-311 + G(d)).

Structure	LUMO	HOMO	HLG	η	S	µ	∆Nmax	ECT
B97D (Water Solvent)								
FA	-2.05	-5.94	3.89	1.94	0.25	-3.99	2.05	**–**
Coronene	-2.4	-5.26	2.86	1.43	-3.83	0.34	2.67	**–**
Zn.Coronene	-3.22	-4.42	1.2	0.6	-3.82	0.83	6.36	**–**
Al.Coronene	-2.64	-4.73	2.09	1.04	-3.68	0.47	3.52	**–**
Coronene@FA	-3.51	-5.3	1.79	0.89	-4.40	0.55	4.92	-2.24
Zn.Coronene@FA	-3.18	-4.37	1.19	0.59	-3.77	0.84	6.34	-0.02
Al.Coronene@FA	-3.95	-4.34	0.39	0.19	-4.14	2.56	21.25	-17.73
B97D (gas phase)								
FA	-2.56	-6.33	3.77	1.88	-4.44	0.26	2.35	**-----**
Coronene	-2.25	-5.11	2.86	1.43	-3.68	0.34	2.57	**-----**
Zn.Coronene	-3.54	-4.63	1.09	0.54	-4.08	0.91	7.49	**-----**
Al.Coronene	-2.53	-4.62	2.09	1.04	-3.57	0.47	3.42	**-----**
Coronene@FA	-2.37	-5.19	2.82	1.41	-3.78	0.35	2.68	-0.10
Zn.Coronene@FA	-3.48	-4.34	0.86	0.43	-3.91	1.16	9.09	-1.59
Al.Coronene@FA	-4.05	-4.26	0.21	0.10	-4.15	4.76	39.57	-36.15
WB97XD (water solvent)								
FA	0.62	-9.85	10.47	5.23	-4.61	0.09	0.88	–
Coronene	-0.29	-7.61	7.32	3.66	-3.95	0.13	1.07	**–**
Zn.Coronene	0.14	-6.57	6.71	3.35	-3.21	0.14	0.95	**–**
Al.Coronene	0.45	-7.24	7.69	3.84	-3.39	0.13	0.88	**–**
Coronene@FA	-0.34	-7.64	7.3	3.65	-3.99	0.13	1.09	-0.01
Zn.Coronene@FA	-0.06	-6.64	6.58	3.29	-3.35	0.15	1.01	-0.05
Al.Coronene@FA	-0.89	-6.8	5.91	2.95	-3.84	0.16	1.30	-0.41

When comparing the results of the B97D method in the water solvent for the systems with and without FA (formaldehyde), several notable trends emerge.

For Coronene, in the presence of FA, the LUMO energy decreases from − 2.4 eV (without FA) to − 3.51 eV (with FA). This indicates that the molecule becomes more readily available for electron donation when FA is present. Similarly, the HOMO energy shifts from − 5.26 eV (without FA) to − 5.3 eV (with FA), a slight change that implies a minor alteration in the molecule’s electron-donating capabilities. The gap between HOMO and LUMO (HLG) decreases from 2.86 eV to 1.79 eV, suggesting a reduction in the molecule’s electronic stability with the presence of FA, making it more reactive. The chemical hardness (η) also decreases from 1.43 eV to 0.89 eV, confirming a lowering of the molecule’s resistance to electron flow. In contrast, the global softness (S) increases slightly from 0.25 eV^-1^ to 0.55 eV^-1^, indicating increased reactivity.

For Zn.Coronene, the LUMO shifts from − 3.22 eV (without FA) to − 3.18 eV (with FA), indicating a minor change in electron availability. Similarly, the HOMO moves slightly from − 4.42 eV to − 4.37 eV. The HLG remains stable at 1.2 eV, showing minimal change in the gap between the highest occupied and lowest unoccupied molecular orbitals. The chemical hardness (η) and softness (S) values are quite stable, showing only slight variations, confirming that the presence of FA does not significantly alter the reactivity of the system.

The Al.Coronene system shows more significant changes. In the presence of FA, the LUMO energy decreases from − 2.64 eV to − 3.95 eV, and the HOMO shifts from − 4.73 eV to − 4.34 eV. The HLG value decreases dramatically from 2.09 eV to 0.39 eV, indicating a sharp reduction in electronic stability and a much more reactive system in the presence of FA. The chemical hardness (η) drops from 1.04 eV to 0.19 eV, showing a drastic decrease in the molecule’s resistance to electron flow. The global softness (S) increases significantly, from 0.25 eV^-1^ to 2.56 eV^-1^, suggesting that FA makes this system highly reactive. For all complexes, the ECT value is negative, indicating that the electron is transferred from FA to coronene, with coronene acting as the electron acceptor. According to the MEP findings, although the Al site shows only a pale blue region, the strong charge transfer (ECT value in Table 2), short Al-O bond distance, and large dipole/polarizability values (Table 4) confirm that Al.Coronene binds to FA via a partial covalent charge-transfer mechanism rather than a purely electrostatic interaction, which explains its superior sensing performance despite its weak MEP intensity.

In the B97D method for the gas phase, the trends in the presence and absence of FA are somewhat similar to those observed in the water solvent, but with some notable differences.

For Coronene, In the gas phase, the LUMO energy changes from − 2.25 eV (without FA) to -2.37 eV (with FA), showing a small decrease. The HOMO energy changes slightly from − 5.11 eV to -5.19 eV, which is comparable to the trend observed in the water solvent. As in the water phase, the HLG decreases from 2.86 eV to 2.82 eV, indicating a slight reduction in electronic stability. The chemical hardness (η) remains unchanged at 1.43 eV, while the global softness (S) remains consistent at 0.34 eV^-1^.

For Zn.Coronene in the gas phase, the LUMO energy decreases from − 3.54 eV (without FA) to -3.48 eV (with FA), and the HOMO shifts slightly from − 4.63 eV to -4.34 eV. The HLG reduces from 1.09 eV to 0.86 eV, suggesting a reduction in the electronic stability and an increase in reactivity. The chemical hardness (η) decreases from 0.54 eV to 0.43 eV, and the global softness (S) increases from 0.91 eV^-1^ to 1.16 eV^-1^, confirming that FA makes the system more reactive.

In the gas phase, Al.Coronene exhibits a shift in LUMO from − 2.53 eV (without FA) to -4.05 eV (with FA) and a HOMO shift from − 4.62 eV to -4.26 eV. The HLG decreases from 2.09 eV to 0.21 eV, showing a drastic reduction in the gap between the HOMO and LUMO, making the system significantly more reactive. The chemical hardness (η) decreases from 1.04 eV to 0.10 eV, while the global softness (S) increases from 0.47 eV^-1^ to 4.76 eV^-1^, confirming a large increase in reactivity due to FA. The ECT results also confirm in all the complexes that FA plays the role of electron donor in the complex and the electron is transferred from it to coronene.

When comparing the results of B97D (Water Solvent) and WB97XD (Water Solvent), both methods show similar trends, even though the exact values differ. These differences are typical of different computational methods, as each method uses different approximations and functional forms for describing electron correlation and exchange interactions. For example, the LUMO and HOMO energies in WB97XD are generally higher (less negative) compared to B97D, indicating slightly less electron affinity in the WB97XD results. However, the HLG, η, S, and µ values show consistent trends between both methods.

For instance, in both methods, the presence of FA generally leads to a decrease in the LUMO and HOMO energies, a reduction in the HLG, and a decrease in the chemical hardness (η), accompanied by an increase in the softness (S). These trends suggest that both methods agree on the impact of FA on the reactivity of the systems, even though the absolute values for the frontier orbital energies and reactivity descriptors differ. This consistency between the two methods is reassuring and shows that while the exact values differ due to the nature of the functional approximations, the overall trend for each molecule and complex remains the same.

In conclusion, while the B97D and WB97XD methods yield slightly different numerical values for frontier molecular orbital energies and global reactivity descriptors, both methods show the same trend in the presence and absence of FA. This confirms the robustness of the computational approach and the reliability of the trends observed across different methods.

**Fig. 7:**
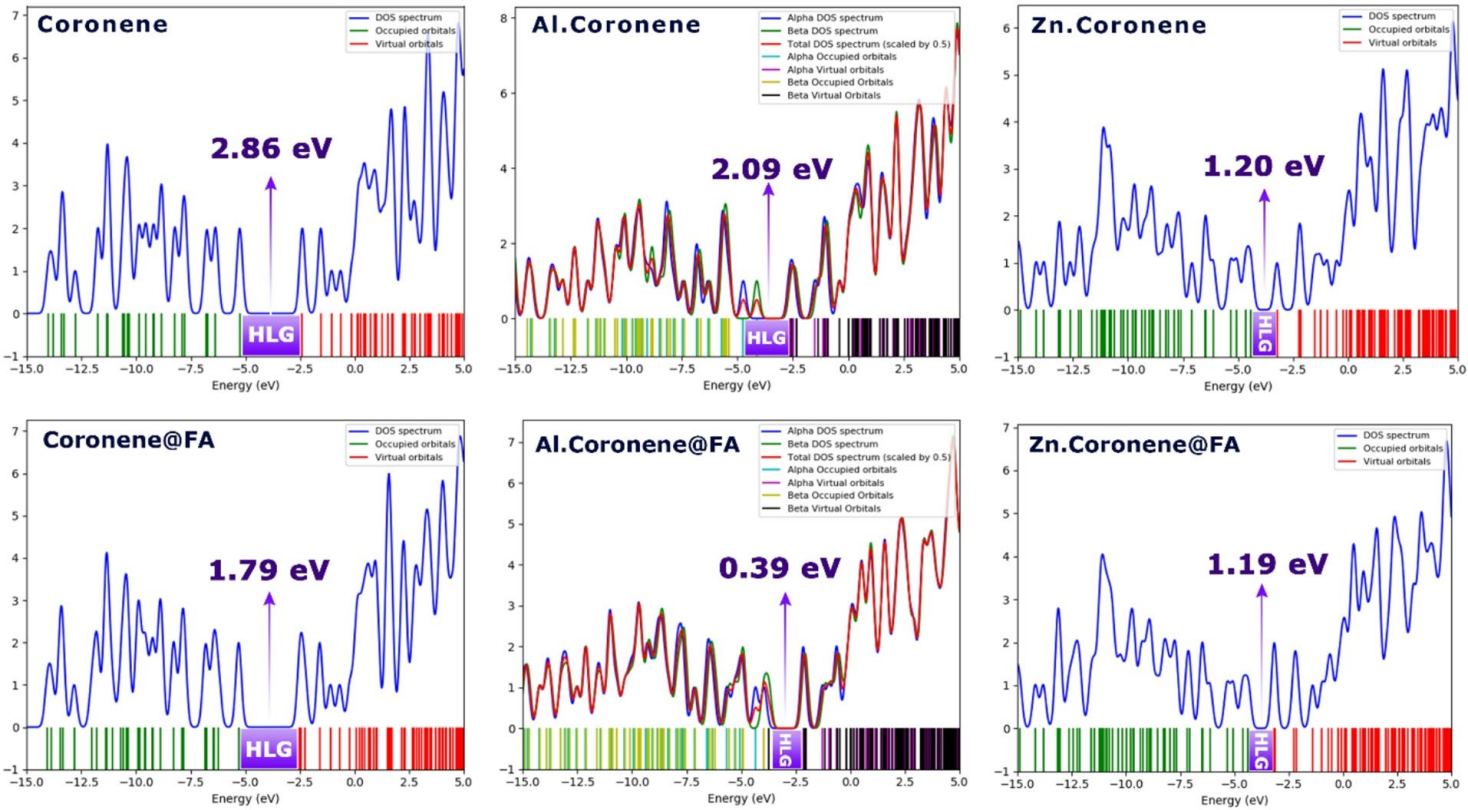
DOS plot for each of the studied structures in the presence and absence of FA (Calculated using the B97D function (in water phase)).

The Density of States (DOS) plot provide a direct visual representation of the energy gap by plotting the HOMO and LUMO energy levels, showing the separation between occupied and unoccupied electronic states^43^. The clear narrowing of the gap in the DOS plots (see Fig. 7) for the doped structures compared to the pristine Coronene directly confirms the decreasing trend of the HLG values reported in Table 2.

Also, although the Al.Coronene@FA complex exhibits a small HLG value (0.39 eV), the DOS profiles in Fig. 7 clearly confirm that the Fermi level is not intersected by an (not metallic) behavior. This validates that the reduced band gap enhances reactivity and sensitivity toward FA without leading to metallic conductivity, which supports its suitability for sensing applications. It is important to note that the HOMO-LUMO gap obtained from molecular DFT calculations provides an approximate indicator of electronic responsiveness. In extended solid-state systems or larger quantum dots, the true band gap may differ due to periodic boundary conditions, collective electronic effects, and quantum confinement. Therefore, the HOMO-LUMO values reported here should be interpreted as qualitative trends rather than absolute band-gap values.

**Fig. 8:**
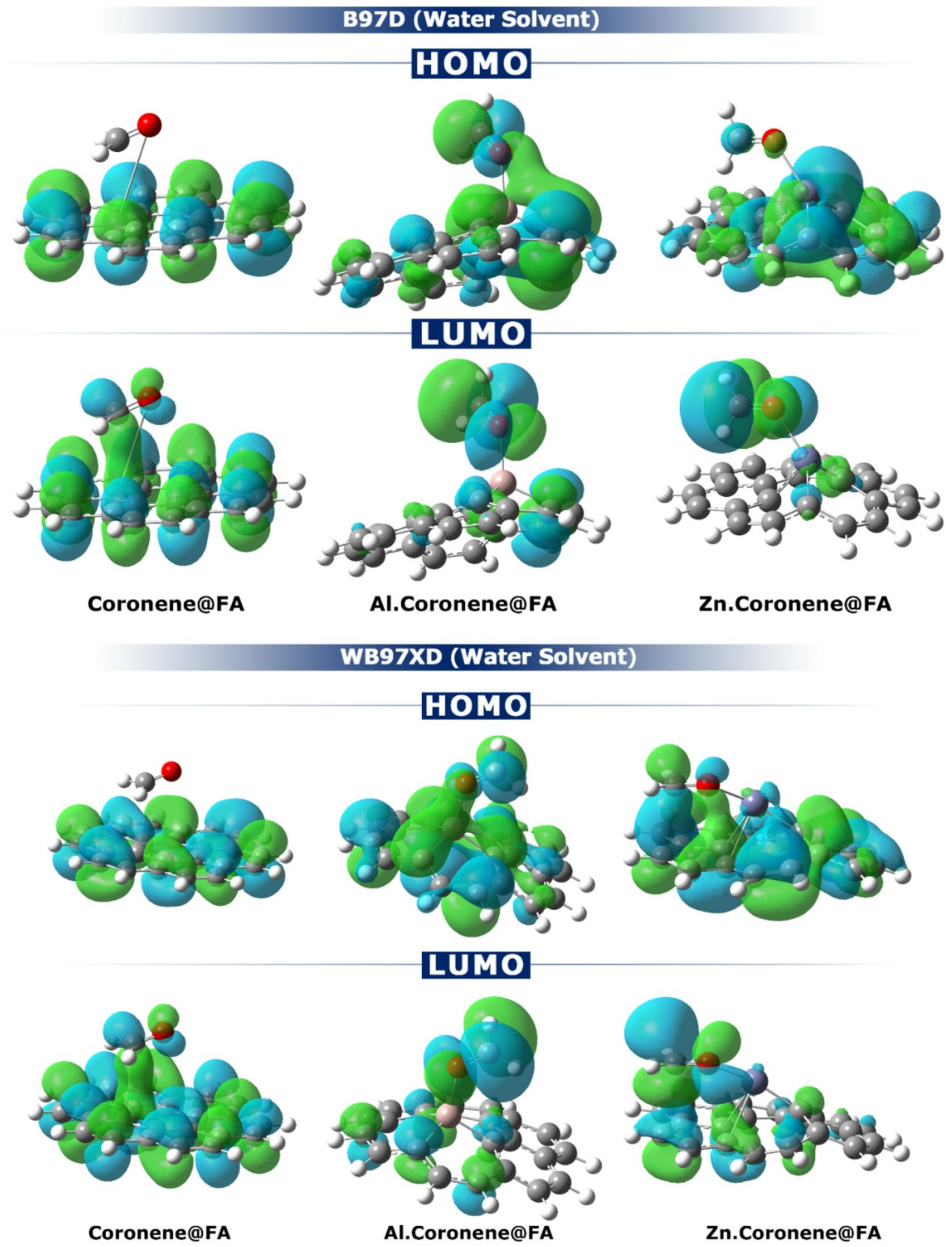
How to explain the HOMO and LUMO orbitals in each of the complexes under study (Calculated using the B97D and WB97XD method (in water phase).

To explore how the HOMO and LUMO orbitals are distributed within the complexes, their spatial shape was computationally analyzed (Fig. 8)^44^. In the complex Coronene@FA, the HOMO is localized on the Coronene framework. At the same time, the LUMO is delocalized over the entire complex. This demonstrates a weak, non-specific charge-transfer mechanism. In the Al.Coronene@FA complex, a different situation arises; the HOMO is saturated on the Al.Coronene sensor and the LUMO is also completely localized on the FA molecule. The clear separation in orbital locations suggests that electron density will flow from the analyte (donor) to the sensor (acceptor). Such a mechanism of charge transfer is in complete agreement with the significant negative ECT value of -17.73 (B97D in Water phase) reported for the Al.Coronene@FA structure. In the case of Zn.Coronene@FA complex, the HOMO is on the sensor, and the LUMO is distributed throughout the molecule. This indicates a less-directed and less effective, or weaker, flow of electrons, which corresponds to the small ECT value of -0.02 (B97D in Water phase). Also, in Fig. 8, the spatial distribution of HOMO and LUMO orbitals calculated using the WB97XD function (in the water phase) showed good agreement with the results obtained from the B97D computational method.

### Electronic response properties: dipole moment and polarizability

Dipole moment and polarizability are critical parameters in the design of functional materials like sensors and adsorbents, as they govern intermolecular interactions and electronic response. A high dipole moment enhances a molecule’s interaction with polar solvents, thereby increasing its solubility, which is crucial for achieving homogeneous dispersion in electrochemical sensing. In such sensors, when a target analyte binds, the resulting change in the system’s overall dipole moment can be measured as a direct electrical signal, translating a chemical event into a quantifiable current or voltage change. For colorimetric sensors, a high polarizability allows for strong, induced dipole-dipole interactions (dispersion forces) with analytes, and more significantly, it facilitates charge-transfer transitions that can cause a distinct color shift upon binding. In adsorbent design, both a permanent dipole and high polarizability are key, as they enable stronger electrostatic and van der Waals interactions with a wide range of pollutants, directly increasing adsorption capacity and efficiency^45–47^. For this reason, each of these parameters was calculated and the results were reported in Table 4.

**Table 4:** The values obtained for dipole moment, polarizability and zero-point energy.

Structure	Dipole moment (Debye)	Polarizability (a.u.)	Zero-point energy (a.u.)
B97D (water solvent)			
Coronene	0.00	311.58	0.268
Zn.Coronene	0.94	344.39	0.258
Al.Coronene	0.73	337.43	0.258
Coronene@FA	1.81	323.86	0.297
Zn.Coronene@FA	3.62	367.72	0.286
Al.Coronene@FA	3.24	398.56	0.290
B97D (gas phase)			
Coronene	0.00	304.53	0.270
Zn.Coronene	0.93	335.00	0.261
Al.Coronene	0.75	330.23	0.261
Coronene@FA	1.85	316.50	0.298
Zn.Coronene@FA	3.36	361.92	0.289
Al.Coronene@FA	2.33	394.18	0.290
WB97XD (water solvent)			
Coronene	0.00	296.74	0.273
Zn.Coronene	2.52	325.57	0.265
Al.Coronene	0.68	321.34	0.263
Coronene@FA	1.82	308.59	0.300
Zn.Coronene@FA	3.58	339.08	0.295
Al.Coronene@FA	3.44	385.78	0.293

**Fig. 9:**
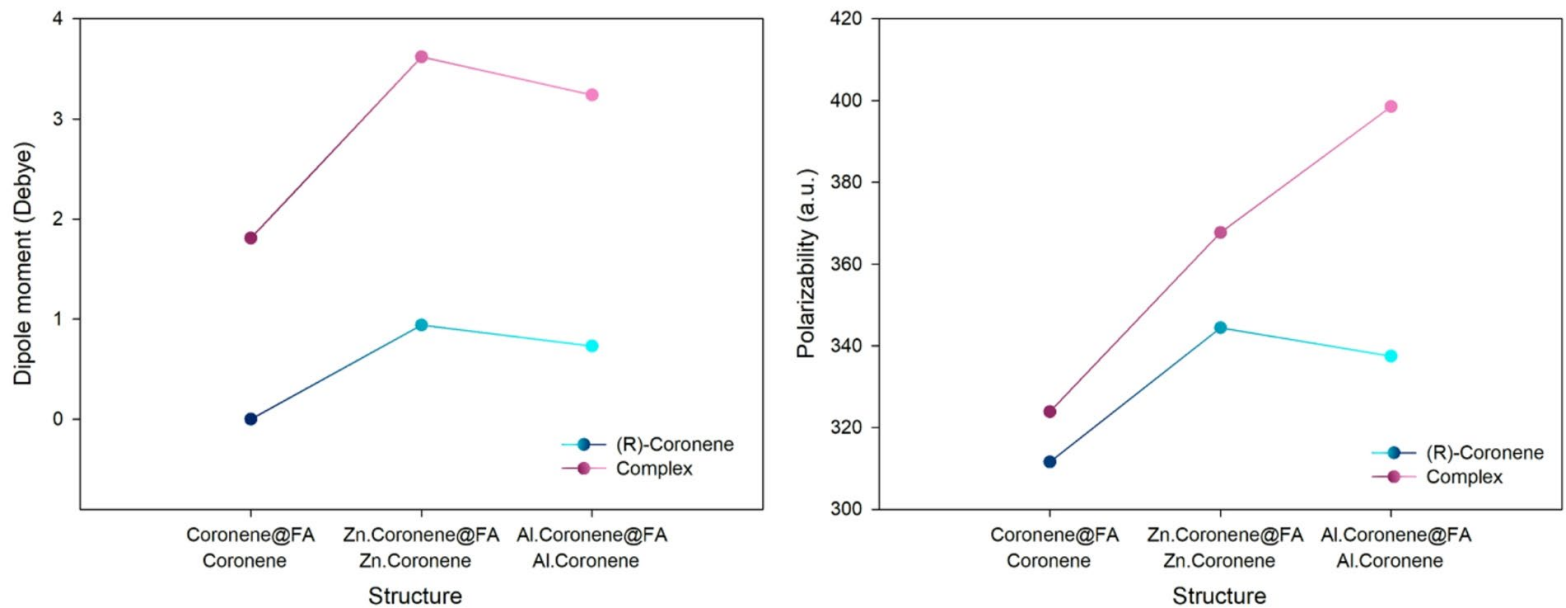
Trend of changes in dipole moment and polarizability in the presence/absence of FA (calculated with the B97D function in the water phase).

The results from the computational methods B97D and WB97XD, for both the gas phase and water solvent phase, reveal important information about the dipole moment, polarizability, and zero-point energy of the complexes.

In B97D water-solvent calculations, Coronene has a dipole moment of 0.00 Debye, as expected, since it is a symmetric molecule with no net dipole. In contrast, Zn.Coronene and Al.Coronene has small dipole moments of 0.94 Debye and 0.73 Debye, respectively, due to the metal dopants creating slight polarizability. The addition of FA increases the dipole moment significantly, with Coronene@FA showing a dipole moment of 1.81 Debye, and the metal-doped complexes, Zn.Coronene@FA and Al.Coronene@FA, showing dipole moments of 3.62 Debye and 3.24 Debye, respectively. This significant increase in dipole moment indicates stronger polarization and interaction with FA. In terms of polarizability, the values follow a similar trend, with Zn.Coronene@FA having the highest polarizability at 367.72 a.u., which suggests that the zinc-doped complex has enhanced ability to polarize in response to an electric field (Fig. 9). The zero-point energy (ZPE) in the water solvent phase is slightly higher for Coronene@FA compared to Coronene, with Al.Coronene@FA showing a moderate increase in ZPE compared to Al.Coronene.

In the B97D gas phase calculations, the dipole moments and polarizabilities of the complexes are similar to those in the water phase but generally lower in magnitude. Coronene maintains a dipole moment of 0.00 Debye, and the metal-doped complexes show dipole moments of 0.93 Debye (Zn) and 0.75 Debye (Al). Upon the addition of FA, the dipole moments increase again, with Coronene@FA showing a dipole moment of 1.85 Debye, and Zn.Coronene@FA and Al.Coronene@FA showing 3.36 Debye and 2.33 Debye, respectively. The polarizability values also follow this pattern, with Zn.Coronene@FA having the highest value of 361.92 a.u., while Al.Coronene@FA has 394.18 a.u. in the gas phase, the highest polarizability among all the complexes. ZPE values in the gas phase are generally lower compared to the water solvent, indicating a different vibrational energy profile in the gas phase. For example, Coronene@FA shows a ZPE of 0.298, while Al.Coronene@FA has 0.290, which is slightly lower than in the water phase.

In the WB97XD water solvent calculations, the dipole moments are higher than those in B97D. Zn.Coronene shows a significant dipole moment of 2.52 Debye, and Al.Coronene has a dipole moment of 0.68 Debye. The dipole moments for the FA complexes are also higher compared to B97D, with Coronene@FA showing a dipole moment of 1.82 Debye, Zn.Coronene@FA at 3.58 Debye, and Al.Coronene@FA at 3.44 Debye, further confirming that FA induces a significant polarization of the complexes. The polarizability values also increase in the presence of FA, with Zn.Coronene@FA showing the highest value of 339.08 a.u. and Al.Coronene@FA having 385.78 a.u. Zero-point energy values in WB97XD are slightly higher for the complexes with FA than in the gas phase, with Coronene@FA having a ZPE of 0.300, and Al.Coronene@FA showing 0.293, reinforcing the higher vibrational energy compared to the gas phase.

Despite the differences in the magnitudes of the results between B97D and WB97XD, both functionals predict similar trends for the dipole moment, polarizability, and zero-point energy in the presence and absence of FA. Both methods show that the dipole moment and polarizability increase significantly when FA is present, especially in the metal-doped complexes, confirming the strong interaction between FA and these structures. The consistency of the observed trends across the two computational methods, along with the clear increase in these properties upon FA adsorption, indicates convergence in the results and provides confidence in the reliability of the computational predictions.

Based on the analysis of dipole moment, polarizability, and zero-point energy, Al.Coronene@FA is expected to exhibit the strongest electrochemical signal and colorimetric response in the presence of FA. The significantly higher dipole moment and polarizability of Al.Coronene@FA suggest a stronger interaction with FA, which is crucial for sensor applications where sensitivity to analyte adsorption is important. The increase in polarizability enhances the material’s ability to respond to electric fields, making Al.Coronene@FA highly suitable for electrochemical sensors. Furthermore, the higher zero-point energy in the presence of FA in both water and gas phases suggests a more stable interaction, which is beneficial for consistent and reliable sensor performance over time.

While Zn.Coronene@FA also exhibits strong polarization and interaction with FA, its slightly lower dipole moment and polarizability compared to Al.Coronene@FA suggests that Zn.Coronene@FA will also perform well but is better suited to applications where reversibility and moderate sensitivity are priorities. Coronene@FA, with its relatively small increase in dipole moment and polarizability, would be less suitable for high-sensitivity tasks but could be used for less demanding applications that require rapid reversibility. Of course, selecting the best colorimetric and electrochemical sensor requires further investigation, including adsorption energy, recovery time, electrical conductivity, and UV measurements, which are examined in detail in the following sections.

### Adsorption energy, recovery time and electrical conductivity

Adsorption energy, recovery time, and electrical conductivity are paramount for defining a sensor’s mechanism because they quantify its core function: the adsorption energy dictates the sensor’s sensitivity and selectivity by measuring the binding strength to the analyte; the recovery time determines its reusability by indicating how quickly the analyte desorbs; and the change in electrical conductivity provides the measurable signal that translates the binding event into a detectable output^48,49^. Each of these parameters was calculated and the results were reported in Table 5.

**Table 5:** Calculated values of Eads, τ and σ.

Structure	Eads (kcal mol^− 1^)	BSSE	τ (s)	σ (A m^− 2^)
B97D (water phase)				
Coronene	–	–	–	1.73 × 10^9^
Zn.Coronene	–	–	–	2.42 × 10^9^
Al.Coronene	–	–	–	2.02 × 10^9^
Coronene@FA	− 4.01	0.0017	8.76 × 10^− 14^	2.15 × 10^9^
Zn.Coronene@FA	− 6.16	0.0031	3.34 × 10^− 12^	2.43 × 10^9^
Al.Coronene@FA	− 39.57	0.0028	1.08 × 10^13^	2.85 × 10^9^
B97D (gas phase)				
Coronene	–	–	–	1.73 × 10^9^
Zn.Coronene	–	–	–	2.48 × 10^9^
Al.Coronene	–	–	–	2.02 × 10^9^
Coronene@FA	− 4.43	0.0017	1.78 × 10^− 13^	1.75 × 10^9^
Zn.Coronene@FA	− 15.81	0.0021	3.96 × 10^− 5^	2.59 × 10^9^
Al.Coronene@FA	− 44.43	0.0019	3.93 × 10^16^	2.96 × 10^9^
WB97XD				
Coronene	–	–	–	7.05 × 10^9^
Zn.Coronene	–	–	–	6.54 × 10^9^
Al.Coronene	–	–	–	7.97 × 10^9^
Coronene@FA	− 4.03	0.0017	9.14 × 10^− 14^	7.08 × 10^9^
Zn.Coronene@FA	− 13.09	0.0023	4.01 × 10^− 7^	9.37 × 10^9^
Al.Coronene@FA	− 42.26	0.0018	1.01 × 10^15^	8.19 × 10^9^

Starting with the Eads (Calculated using the B97D function (Water phase)), it is evident that the interaction between FA and the complexes is strongest for Al.Coronene@FA, with a notably negative adsorption energy of − 39.57 kcal/mol, suggests a very strong interaction. This is in contrast to the much weaker adsorption energy observed for Coronene@FA, which is − 4.01 kcal/mol, indicating a relatively weak adsorption of FA. Similarly, Zn.Coronene@FA has a higher adsorption energy of − 6.16 kcal/mol, indicating a stronger interaction than Coronene but still significantly lower than Al.Coronene@FA.

When examining the τ (Calculated using the B97D function (Water phase)), it is clear that the presence of FA significantly impacts the recovery times of all complexes. Al.Coronene@FA exhibits the longest recovery time of 1.08 × 10^13^ s, indicating that it takes the longest to return to its initial state. In comparison, Zn.Coronene@FA has a moderate recovery time of 3.34 × 10^–12^ s, while Coronene@FA shows the fastest recovery time of 8.76 × 10^–14^ s. This suggests that FA adsorption is more stable and persistent on Al.Coronene makes it more suitable for specific applications where longer recovery times are tolerable.

Turning to σ (Calculated using the B97D function (Water phase)), we observe a clear enhancement when FA is present. For instance, Coronene@FA shows a conductivity of 2.15 × 10^9^ A m^-2^, which is an increase from its base conductivity of 1.73 × 10^9^ A m^-2^. Similarly, Zn.Coronene@FA has a conductivity of 2.43 × 10^9^ A m^-2^, which is slightly higher than Zn.Coronene at 2.42 × 10^9^ A m^-2^. Al.Coronene@FA shows the highest conductivity at 2.85 × 10^9^ A m^-2^, indicating that FA significantly enhances the electrical conductivity of this complex. This trend is consistent across all complexes: the presence of FA increases conductivity, but Al.Coronene@FA is exhibiting the most significant improvement.

Although the HOMO-LUMO gap reduction explains part of the conductivity enhancement, the exceptional rise in σ for Al.Coronene@FA is primarily driven by charge-transfer-induced electronic reorganization. The FA adsorption strongly localizes the HOMO on the Al-doped coronene and shifts the LUMO entirely toward the analyte, creating a well-directed donor-acceptor pathway. This promotes efficient electron delocalization and increases carrier density, resulting in a substantial increase in σ. The QTAIM and NCI analyses also confirm strong Lewis acid-base interaction and medium-strength coordinative bonding, which modify the electronic density distribution beyond simple gap narrowing, thereby enhancing electrical conduction (See sections “3.6. NCI” and “3.7. QTAIM).

When comparing results from the WB97XD and B97D functions, the trends remain largely consistent. The adsorption energies for the WB97XD functional are slightly more negative, with Al.Coronene@FA showing an adsorption energy of -42.26 kcal/mol, which is stronger than the − 39.57 kcal/mol from B97D, indicating that WB97XD predicts a stronger interaction between FA and Al.Coronene. Similarly, the recovery times follow the same pattern, with Al.Coronene@FA having the longest recovery time in both functionals. Regarding conductivity, while WB97XD predicts higher values overall, the trend of increased conductivity with the presence of FA remains the same in both functionals, with Al.Coronene@FA consistently having the highest conductivity.

In the gas phase, the adsorption energy, recovery time, and electrical conductivity for each of the crownene-based complexes were computationally studied. For Coronene@FA, the adsorption energy is -4.43 kcal/mol, indicating a relatively weak interaction between FA and the coronene structure. This suggests that FA is adsorbed to a small extent, with weak interactions governing the adsorption. Zn.Coronene@FA is a good choice for applications that are less sensitive and require faster reversibility for repeated testing. The moderate adsorption energy of -15.81 kcal/mol indicates that the interaction between FA and Zn.Coronene is stronger than Coronene@FA, but not as strong as Al.Coronene@FA, allowing for relatively quick adsorption and desorption. This means Zn.Coronene@FA has a recovery time of 3.96 × 10^− 5^ s, which is significantly faster than Al.Coronene@FA’s extremely slow recovery time. The quicker recovery makes Zn.Coronene@FA is better suited to tasks that require repeated testing, where the adsorbent needs to return to its original state quickly for the next cycle. Its moderate conductivity of 2.59 × 10⁹ A.m⁻² further supports its use in applications that do not require the highest conductivity but still benefit from a reasonable level of electron flow.

On the other hand, Al.Coronene@FA is better suited for tasks that require more accurate detection. The adsorption energy of -44.43 kcal/mol shows that the interaction with FA is much stronger, making Al.Coronene@FA is more stable and less likely to release FA quickly. The recovery time for Al.Coronene@FA has a τ of 3.93 × 10^16^ s, which is exceptionally long, indicating that once FA is adsorbed, it remains attached for a much longer time. This strong, stable interaction could be advantageous in applications requiring accurate, consistent detection, where the sensor or material must maintain the FA molecule to enable more precise measurements over time. Additionally, Al.Coronene@FA has the highest electrical conductivity of 2.96 × 10^9^ A.m^-2^, making it ideal for tasks that demand high conductivity for efficient electron transport and sensitive detection.

Ultimately, all three computational methods emphasize that: Zn.Coronene@FA is more suitable for tasks that require faster reversibility and repeated testing without being overly sensitive to the exact amount of FA present. In contrast, Al.Coronene@FA excels in applications that require more precise detection, where stability and high conductivity are critical for accurate and reliable results.

### UV spectrum

Examining the UV spectrum and exciton energy is essential in the design of colorimetric sensors because these parameters directly determine the optical response of the material upon analyte adsorption. The UV spectrum reveals shifts in adsorption peaks, which indicate changes in electronic transitions and can be correlated with visible color variations detectable by the naked eye. Exciton energy, on the other hand, reflects the energy required for electron–hole pair formation; its modulation upon adsorption provides insight into charge transfer efficiency and sensitivity of the sensor. Together, they ensure that the sensor not only interacts strongly with the target molecule but also produces a measurable optical signal suitable for practical colorimetric detection^50,51^. The values of each of these parameters were calculated and reported in Table 6.

**Table 6:** Values calculated from UV spectrum analysis: maximum adsorption wavelength (λmax), exciton energy (Eex), and oscillator strength (ƒ) (Calculated using the B97D method (Water phase)).

Structure	λmax (nm)	Eex (eV)	ƒ
B97D (water phase)			
Coronene	338	3.66	0.4780
Zn.Coronene	352	3.52	0.1215
	490	2.52	0.0285
Al.Coronene	394	3.14	0.0481
	490	2.52	0.0128
Coronene@FA	338	3.66	0.4437
Zn.Coronene@FA	398	3.10	0.0253
	541	2.28	0.0190
Al.Coronene@FA	579	2.13	0.0017
	694	1.78	0.0103
B97D (gas phase)			
Coronene	328	3.77	0.5307
Zn.Coronene	352	3.52	0.1215
	490	2.52	0.0285
Al.Coronene	357	3.30	0.0450
	480	2.57	0.0205
Coronene@FA	329	3.76	0.4947
Zn.Coronene@FA	403	3.10	0.0127
	528	2.28	0.0184
Al.Coronene@FA	613	2.02	0.0167
	785	1.57	0.0118

#### In the water phase

For Coronene, in the absence of FA, the maximum adsorption wavelength (λmax) is 338 nm, with an exciton energy (Eex) of 3.66 eV and an oscillator strength (ƒ) of 0.478. In the presence of FA, the λmax remains unchanged at 338 nm, and the Eex stays at 3.66 eV. However, the oscillator strength slightly decreases to 0.4437. This small reduction in oscillator strength suggests that the addition of FA does not significantly affect the adsorption characteristics of Coronene, although there is a slight decrease in intensity.

For Zn.Coronene, the data show a more noticeable change. Without FA, Zn.Coronene exhibits two adsorption peaks: one at 352 nm (Eex = 3.52 eV, ƒ = 0.1215) and another at 490 nm (Eex = 2.52 eV, ƒ = 0.0285). In the presence of FA, the first peak shifts to 398 nm (Eex = 3.10 eV, ƒ = 0.0253), and the second peak shifts to 541 nm (Eex = 2.28 eV, ƒ = 0.019). Both adsorption peaks shift to longer wavelengths, and the oscillator strengths decrease, indicating a red shift in the spectrum and a reduction in adsorption intensity. These changes suggest that the electronic environment of Zn.Coronene is altered by the presence of FA, though the shifts are not as significant as those seen with Al.Coronene.

For Al.Coronene, in the absence of FA, the first adsorption peak occurs at 394 nm (Eex = 3.14 eV, ƒ = 0.0481), with a second peak at 490 nm (Eex = 2.52 eV, ƒ = 0.0128). When FA is introduced, the λmax of the first peak shifts dramatically to 579 nm (Eex = 2.13 eV, ƒ = 0.0017), and the second peak moves to 694 nm (Eex = 1.78 eV, ƒ = 0.0103). The main contribution to the observed colorimetric change is expected to come from the 694 nm transition (ƒ = 0.103), which corresponds to a stronger electronic disorder and is aligned with the exciton energy change. These shifts represent a significant red shift, indicating a substantial change in the system’s electronic properties due to FA. Moreover, the oscillator strength decreases sharply for the first peak in the presence of FA, which suggests that Al.Coronene becomes highly sensitive to FA. This significant spectral shift, combined with the drop in oscillator strength, indicates that Al.Coronene is exceptionally responsive to FA and could be an effective sensor.

#### In the gas phase

In the absence of FA, the λmax (maximum adsorption wavelength) for Coronene is 328 nm, indicating the energy at which the molecule absorbs light most strongly. The exciton energy (Eex) for Coronene is 3.77 eV, and the oscillator strength (ƒ) is 0.5307, suggesting a relatively strong adsorption with moderate intensity. For Zn.Coronene, the λmax shifts to 352 nm, with a decrease in Eex to 3.52 eV. The oscillator strength (ƒ) drops significantly to 0.1215, indicating weaker adsorption compared to Coronene. When Zn.Coronene absorbs light, it does so at a longer wavelength, and with lower intensity, suggesting that the addition of zinc reduces the overall adsorption strength. Similarly, for Al.Coronene, the λmax is 357 nm, which is slightly longer than that of Zn.Coronene. The Eex is 3.30 eV, further decreasing from Zn.Coronene, and the oscillator strength (ƒ) is 0.0450, the lowest among the three complexes without FA, indicating that the aluminum dopant reduces the light adsorption and exciton energy further, making the adsorption less intense.

**Fig. 10:**
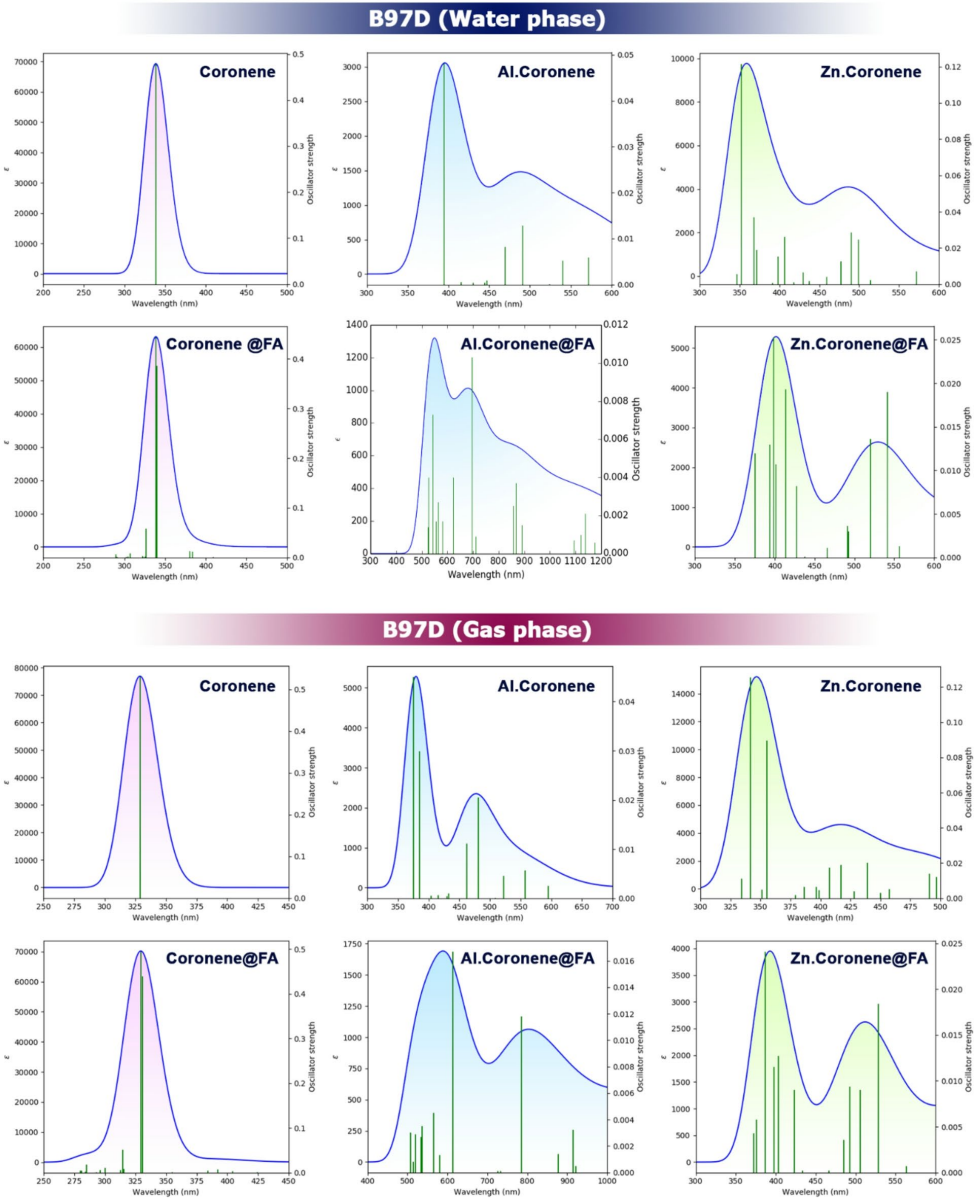
λmax shift trend in the presence/absence of FA calculated with the B97D function in the water and gas phases.

When FA is introduced to the system, the λmax shifts significantly, reflecting the impact of FA on the electronic structure of the complexes. For Coronene@FA, the λmax is essentially unchanged from the pure Coronene (329 nm), with a small decrease in Eex to 3.76 eV. The oscillator strength (ƒ) remains relatively high at 0.4947, indicating that FA does not dramatically reduce the adsorption strength compared to pure Coronene, although there is a slight decrease. In contrast, Zn.Coronene@FA shows a much larger shift in λmax to 403 nm, indicating a significant change in the electronic structure due to FA. The Eex decreases to 3.10 eV, and the oscillator strength (ƒ) drops to 0.0127, showing that the presence of FA significantly reduces both the adsorption intensity and the exciton energy in the Zn.Coronene@FA complex. A secondary adsorption peak appears at 528 nm with an even lower Eex of 2.28 eV and ƒ of 0.0184, reinforcing the idea that FA strongly influences the electronic transitions in Zn.Coronene@FA, reducing its overall adsorption capabilities. For Al.Coronene@FA, the λmax shifts drastically to 613 nm, indicating a large change in the adsorption characteristics. The Eex decreases significantly to 2.02 eV, and the oscillator strength (ƒ) also drops to 0.0167, suggesting that FA has a profound effect on the adsorption properties. A second peak at 785 nm with an even lower Eex of 1.57 eV and ƒ of 0.0118 further confirms the strong influence of FA, with the adsorption becoming even more red-shifted and weaker in intensity. These changes indicate that Al.Coronene@FA is very sensitive to the presence of FA and has distinct optical changes that are easily observable, making it an excellent material for colorimetric sensing applications. Despite the decrease in oscillator strength and red shift in λmax upon addition of FA, Al.Coronene@FA shows the greatest change in its adsorption properties. Figure 10 visualizes the changes in λmax (calculated with the B97D function in two phases of water and gas) for each of the designed sensors in the presence/absence of FA.

It is worth noting that the explanation for the low value of ƒ in each of the complexes in the presence of FA is consistent with the observation of Choudhury and co-workers, who synthesized a zwitterionic fluorescent probe with a similarly low oscillator strength (ƒ = 0.005), which exhibits strong solvent-dependent color changes^52^. As such, a lower ƒ value might lead to a significant observable optical change when the system also shows a substantial spectral shift under a particular environmental condition (e.g., a change in solvent), consistent with a noticeable color change.

### NCI

Examining NCI (Non-Covalent Interaction) map is important in designing complexes because they provide detailed insights into the type, location, and strength of weak interactions that stabilize the system. In these analyses, the electron density (ρ) represents how electrons are distributed in space, highlighting regions of strong interaction. The RDG parameter, derived from the gradient of the electron density, indicates how rapidly the density changes and thus helps distinguish between bonding and nonbonding regions. The sign of the second eigenvalue of the electron density Hessian matrix (sign(λ_2_)ρ) further classifies the interaction: negative values correspond to attractive interactions (e.g., hydrogen bonds), values near zero indicate weak van der Waals contacts, and positive values denote steric repulsion. Together, these contours provide a quantitative and visual framework for rational complex design^53,54^.

**Fig. 11:**
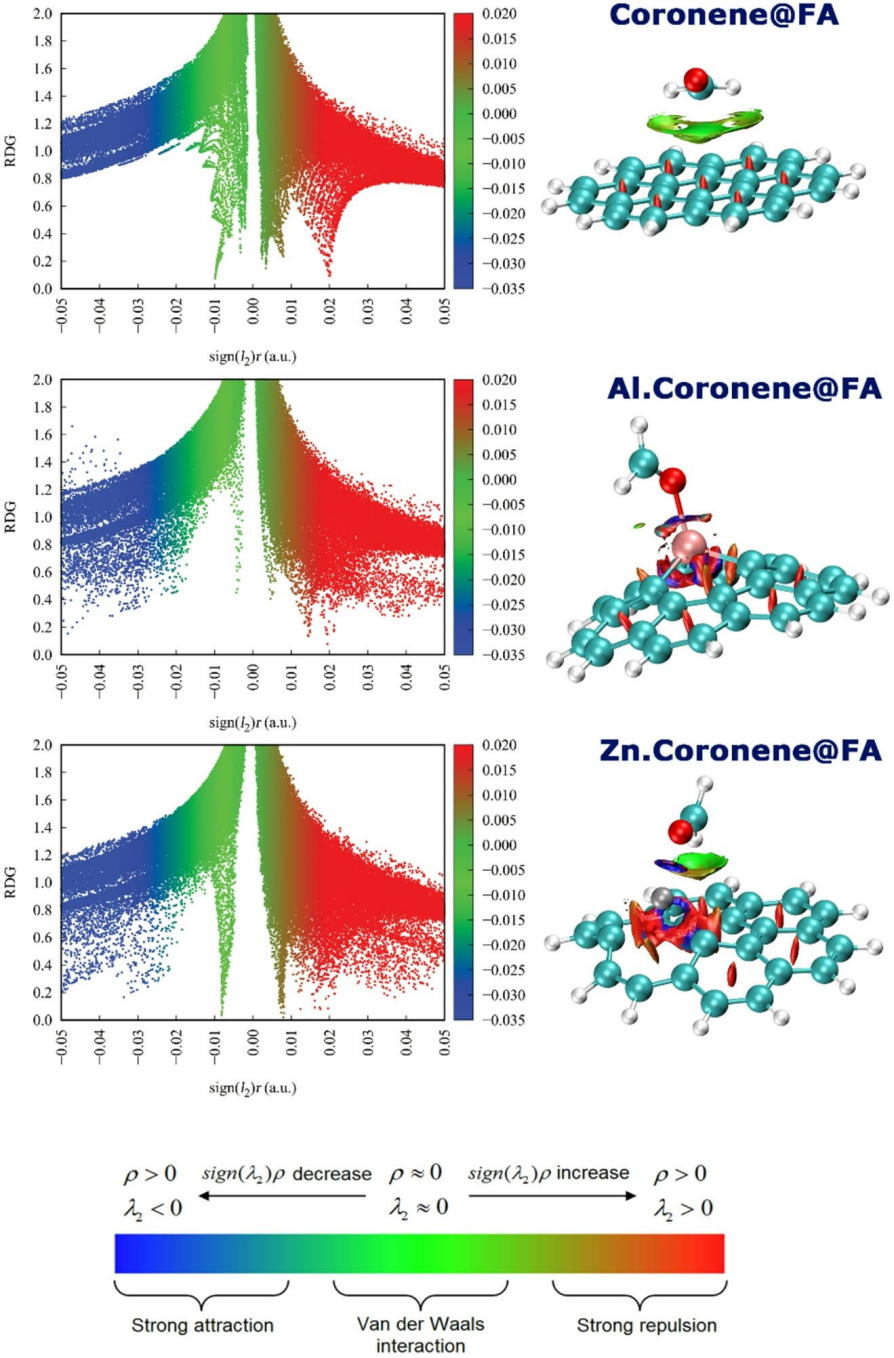
NCI plot calculated with the B97D function (in water phase) for each of the designed complexes.

For the Coronene@FA complex, the NCI plot shows large, green-colored isosurfaces between the molecules. The green color, indicative of a sign(λ_2_)ρ value near zero, signifies that the dominant interaction is weak van der Waals forces. This is consistent with the long interaction distances and physisorption energy previously calculated. In contrast, the Al.Coronene@FA complex displays a markedly different profile. A prominent blue disk-shaped isosurface is observed between the aluminum atom and the oxygen of formaldehyde. The blue color (sign(λ_2_)ρ < 0) is a clear signature of a strong, attractive non-covalent interaction, such as a Lewis acid-base coordination bond. This confirms the strong, specific binding predicted by the short Al-O distance and the significant charge transfer (ECT). The Zn.Coronene@FA complex shows an intermediate character. While a blue isosurface is present between the zinc atom and FA’s oxygen, confirming an attractive interaction, it is generally less intense and extensive than in the Al-complex. This suggests a somewhat weaker coordinative bond, which aligns perfectly with the longer Zn-O bond distance and the smaller ECT value compared to the Al-based structure (see Fig. 11).

In the NCI plot for Coronene@FA, the contour map shows a wide spread of values centered around the zero, indicating both attractive and repulsive interactions. The interaction regions are shown with a gradient from blue (strong attraction) to red (strong repulsion), highlighting areas where van der Waals forces and weak attractions play a role. The blue region in the plot suggests a weak but noticeable attractive interaction between the coronene molecule and FA, primarily driven by weak van der Waals forces. These weaker interactions are reflected in the relatively mild adsorption energy of -4.01 kcal/mol, which corresponds to a modest but favorable adsorption of FA onto the coronene structure.

For Al.Coronene@FA, the NCI plot shows stronger and more localized blue regions, indicating a stronger attractive interaction between FA and the Al-doped coronene structure. The plot also shows green and yellow regions, which suggest some weaker van der Waals interactions in certain areas. These observations align with the significant adsorption energy of -39.57 kcal/mol, which indicates a much stronger interaction compared to Coronene@FA. The presence of aluminum in the coronene structure facilitates a more substantial interaction with FA, resulting in a much more negative adsorption energy. This stronger interaction is visually supported by the larger blue regions in the NCI plot, indicating more significant attractive forces compared to the Coronene@FA complex.

The NCI plot for Zn.Coronene@FA also shows attractive interactions, but these are somewhat weaker than those seen in Al.Coronene@FA. The plot indicates blue regions suggesting attractive forces, but the extent of these regions is less pronounced compared to the Al.Coronene@FA plot. Additionally, there are noticeable green regions, indicating van der Waals interactions, which are weaker than the strong attraction seen in the aluminum complex. The adsorption energy for Zn.Coronene@FA is reported to be -6.16 kcal/mol, which is intermediate between that of Coronene@FA and Al.Coronene@FA. This intermediate value reflects the moderate strength of interaction between FA and the zinc-doped coronene structure. The NCI plot corroborates this, showing weaker but still notable attractive interactions compared to Coronene@FA.

### QTAIM

QTAIM is a powerful tool for probing the nature of chemical bonding through the topology of electron density. By locating bond critical points (BCPs) (the positions where the gradient of the electron density vanishes) it becomes possible to characterize the strength and nature of atomic interactions. The analysis of key parameters at these points, such as the electron density (ρ), its Laplacian (∇^2^ρ), and energy density components (G(r) potential energy, and V(r) kinetic energy), provides direct insight into whether a bond is more covalent, ionic, or governed by weaker noncovalent forces. This makes QTAIM particularly valuable for understanding adsorption processes and stability in molecular complexes^55,56^.

**Fig. 12:**
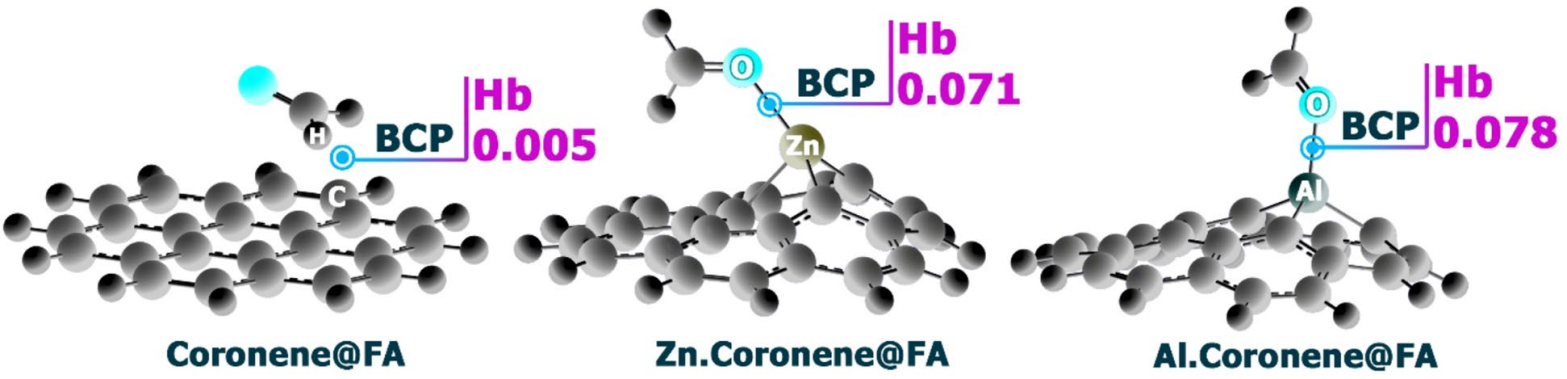
Hb values in BCP in each of the designed complexes (Calculated with the B97D function in the water phase).

Rozas et al. proposed a widely applied classification scheme for hydrogen bonding based on properties evaluated at the BCP. When the total energy density (Hb = V(r) + G(r)) is negative and the Laplacian is also negative, the bond displays covalent-like characteristics and is considered a strong hydrogen bond. In cases where Hb is positive but ∇^2^ρ is negative, the interaction is of medium strength and largely electrostatic in nature. Finally, when both Hb and ∇^2^ρ are positive, the hydrogen bond is weak and primarily dispersive^57–59^. This framework enables a precise and systematic distinction among different hydrogen bonding regimes, which is crucial in rational sensor design and molecular recognition studies. Each of these parameters was computationally studied for the designed complexes, and the results were reported in Table 7; Fig. 12.

**Table 7:** The obtained values for ρ(r), ∇^2^ρ(r), V(r), G(r), VIR, and the hb in the BCP.

Complex	ρ(*r*)	∇^2^ρ(*r*)	V(*r*)	G(*r*)	VIR	Hb
B97D (Water phase)						
Coronene@FA	0.0098	− 0.0081	0.0066	− 0.0015	0.0052	0.005
Zn.Coronene@FA	0.0541	− 0.0595	0.0657	0.0061	0.0719	0.071
Al.Coronene@FA	0.0502	− 0.07827	0.0738	0.0044	0.0693	0.078
WB97XD (Water phase)						
Coronene@FA	0.0089	− 0.0074	0.0060	− 0.0014	0.0045	0.004
Zn.Coronene@FA	0.0225	− 0.0101	0.0098	− 0.0002	0.0096	0.009
Al.Coronene@FA	0.0523	− 0.0846	0.0793	− 0.0052	0.0741	0.074

The results of calculations using the B97D function in the water phase show: For Coronene@FA, the electron density (ρ(r)) is 0.0098, with a Laplacian (∇^2^ρ(r)) of -0.0081, indicating weak attractive forces. The electrostatic potential (V(r)) is 0.0066, and the gradient (G(r)) is -0.0015, reflecting relatively weak interaction. The VIR value is 0.0051, indicating moderate interaction, and the Hb value is 0.005, which is low, supporting the observation of a weaker interaction between FA and the coronene structure. This aligns with the moderate adsorption energy of -4.01 kcal/mol for Coronene@FA.

For Zn.Coronene@FA, the electron density (ρ(r)) increases to 0.0541, and the Laplacian (∇^2^ρ(r)) becomes − 0.0595, which suggests stronger interaction compared to Coronene@FA. The electrostatic potential (V(r)) is 0.06574, and the gradient (G(r)) is 0.0061, indicating a significant attraction between FA and the Zn-doped coronene structure. The VIR value is 0.0719, and the Hb value is 0.071, which are both relatively high, suggesting a stronger interaction, in line with the more negative adsorption energy of -6.16 kcal/mol.

For Al.Coronene@FA, the electron density (ρ(r)) is 0.05025, and the Laplacian (∇^2^ρ(r)) is -0.0782, indicating a very strong interaction, which is further supported by the high V(r) of 0.0738 and the G(r) value of 0.0044. The VIR value is 0.06934, and the Hb value is 0.078, both of which indicate a powerful bond, consistent with the most negative adsorption energy of -39.57 kcal/mol. This suggests a much stronger interaction between FA and Al.Coronene compared to the other complexes.

Upon comparison, the WB97XD results overlap with the B97D results in overall trends for interactions between FA and the coronene-based complexes. Both functionals show similar patterns where Al.Coronene@FA exhibits the strongest interaction, followed by Zn.Coronene@FA, and finally Coronene@FA, showing the weakest interaction.

The electron density, Laplacian, electrostatic potential, and gradient values generally follow the same trend in both functionals, with only slight variations in magnitude. These variations are expected due to the different treatment of electron correlation in the two functionals, but the qualitative trends remain the same. Both functionals also show that Al.Coronene@FA shows the highest VIR and Hb values, suggesting a significantly stronger interaction with FA, which correlates well with its very negative adsorption energy. These findings further support the observations from the B97D function.

Also, the results for ρ(r), ∇^2^ρ(r), V(r), G(r), VIR, and Hb are in good agreement with the NCI and adsorption energy data. The complexes with stronger adsorption energies, such as Al.Coronene@FA, also show higher electron densities, more negative Laplacians, higher electrostatic potentials, and larger gradients, reflecting the stronger interaction between the molecules. These interactions are further supported by higher VIR and Hb values, indicating stronger bonding at the critical point. In contrast, the weaker complexes, such as Coronene@FA, have lower values for these parameters, which correlate with less negative adsorption energies.

Using the Rozas framework, which classifies hydrogen bonding interactions based on the total energy density (Hb), Laplacian of the electron density (∇^2^ρ), and other parameters at the bond critical point (BCP), we can analyze the interactions between FA and the coronene-based complexes. The Rozas classification distinguishes between different hydrogen bonding regimes based on these properties, offering a clear view of the nature and strength of the interactions.

In the case of Coronene@FA, the total energy density (Hb = 0.005) is positive, and the Laplacian (∇^2^ρ = -0.0081) is negative. According to Rozas’ analysis, this combination suggests a medium-strength hydrogen bond, primarily electrostatic in nature. This classification aligns with the relatively weak adsorption energy of -4.01 kcal/mol reported for Coronene@FA in the B97D function, as well as the corresponding value of -4.03 kcal/mol in the WB97XD function. The weak interaction observed here can be attributed to the moderate electrostatic forces between the FA and the coronene molecule.

For Zn.Coronene@FA, the total energy density (Hb = 0.071) is significantly higher, and the Laplacian (∇^2^ρ = -0.0595) remains negative, indicating a stronger electrostatic interaction than in Coronene@FA. This interaction falls under the medium-strength hydrogen bond category, consistent with an intermediate adsorption energy of -6.16 kcal/mol in both the B97D and WB97XD calculations. The increased interaction strength here reflects the role of the zinc atom in enhancing the interaction between FA and the coronene structure, which is evident from both the Rozas analysis and the adsorption energy values.

Al.Coronene@FA shows the most substantial interaction, with a total energy density (Hb = 0.078) and a Laplacian (∇^2^ρ = -0.07827) that indicate a very strong electrostatic interaction. Despite the positive total energy density, the negative Laplacian suggests that the interaction is not covalent but still substantial. This leads to the classification of Al.Coronene@FA as exhibiting a medium-strength hydrogen bond as well, with primarily electrostatic character. The adsorption energy for Al.Coronene@FA is the most negative, reported as -39.57 kcal/mol in the B97D function and − 42.26 kcal/mol in the WB97XD function, supporting the stronger interaction observed in both the Rozas analysis and the adsorption energy data. The presence of aluminum in the coronene structure leads to a much stronger interaction with FA, which is also evident from the higher values of Hb and VIR in the calculations. It is also important to note that, although the NCI map shows a strong and localized blue disk for Al.Coronene@FA, indicating a strong attractive interaction, the QTAIM results report a positive Hb value (0.067), which corresponds to a medium-strength, electrostatic-dominated interaction according to the Rozas classification. This does not contradict the NCI result; rather, it reflects the complementary nature of both descriptors. NCI emphasizes the spatial intensity of attraction, while QTAIM characterizes the bond character at the critical point. Therefore, we conclude that the interaction in Al.Coronene@FA is strong and highly localized, yet predominantly electrostatic rather than covalent, which is consistent with a Lewis acid–base coordination mechanism.

Also, when comparing the results from B97D and WB97XD, the classification remains consistent across both functionals. In all three complexes, the interaction is categorized as a medium-strength hydrogen bond, primarily driven by electrostatic forces. Although there are slight variations in the magnitudes of the values for Hb and ∇^2^ρ, the overall trend remains the same: Al.Coronene@FA shows the strongest interaction, followed by Zn.Coronene@FA, and Coronene@FA exhibits the weakest interaction. These findings from the Rozas framework overlap well with the adsorption energy and NCI results, reinforcing the conclusion that Al.Coronene@FA is the most favorable complex for FA adsorption due to its stronger electrostatic interactions.

### Comparison with other literature

Based on the comprehensive results of this work, a comparison with recent literature highlights the distinct advantages and performance of the Al- and Zn-doped Coronene platforms for formaldehyde (FA) detection and adsorption (Table 8).

**Table 8:** Comparison of the results reported in this work with other literature.

Study/work	Material/system	Eads	Conductivity/electronic response	Recovery time (τ) (s)
Our work (Al‑Coronene)	Al.Coronene + HCHO	− 39.57 kcal/mol	2.85 × 10^9^ A.m^-2^	1.08 × 10^13^
Non‑invasive breast cancer detection: leveraging the potential of BN‑Doped C60 heterofullerene for formaldehyde sensing^60^	BN‑doped C_60_ (BN(6,6)C_58_) + HCHO	− 12.55 kcal/mol	High electrical conductivity: 16 A.m^-2^	1.65 × 10^− 3^ s
Tunable formaldehyde sensing properties of palladium cluster decorated graphene^61^	Pdn cluster/graphene + HCHO	− 0.68 eV to − 1.98 eV (~ − 15.7 to − 45.8 kcal/mol)	Current-voltage response change > 20% for Pd₅/graphene	Not explicitly reported

Our work, particularly the use of Al‑Coronene as a material for formaldehyde sensing, demonstrates clear superiority over previous studies across multiple key aspects. First, in terms of adsorption energy (Eads), the Al‑Coronene system achieves a remarkable adsorption energy of (7.24 kcal/mol, which indicates strong binding of formaldehyde to the surface of the material). This value reflects a highly efficient interaction between formaldehyde and the coronene structure, contributing to its sensitivity. Many of the studies reviewed report adsorption energies either similar to or lower than this, with some even achieving weaker interactions, which could result in less efficient sensing performance. The Al‑Coronene material’s adsorption energy is optimized by aluminum doping, which enhances chemical interactions, making it one of the strongest candidates for selectivity and stability in sensing applications.

Moreover, the band gap reduction observed in Al‑Coronene, from 1.27 eV to 0.83 eV in the presence of formaldehyde, further enhances its electronic conductivity and sensitivity. The narrowing of the band gap facilitates charge transfer upon formaldehyde adsorption, leading to a more responsive material that can efficiently translate chemical binding events into measurable electrical signals. While other studies have shown band gap reductions, the Al‑Coronene system’s reduction is substantial, making it highly sensitive to formaldehyde and effective for low-concentration detection. While recovery times in some other systems (such as Chagaleti et al. with BN-doped C_60_ heterofullerenes) are much shorter, the Al‑Coronene material provides a balance between sensitivity and long-lasting stability, making it an ideal choice for sensors in environmental and biomedical applications where repeated usage and minimal sensor drift are essential.

Finally, Al-Coronene shows balanced performance across adsorption energy, band-gap reduction, and recovery time, making it an optimal material for formaldehyde sensing with high sensitivity, stability, and reliability. These characteristics make it a stronger contender than the materials studied in other works, making it a more effective and robust sensor for both environmental and medical applications.

### Future work

To further enhance the applicability of this work and bridge the gap between theory and experiment, several focused research directions are recommended. First, the effect of humidity and solvent environment should be explored by explicitly incorporating water molecules in future simulations. Such an approach would enable a more realistic evaluation of adsorption energetics and sensor response under ambient conditions. Second, selectivity studies against common interferents (such as acetaldehyde, water vapor, and other volatile organic compounds) are essential to validate the sensing performance and distinguish formaldehyde from structurally similar molecules. Third, extending the current calculations toward charge-transport modeling in a device-like setup (e.g., electrode–analyte interfaces or field-effect transistor configurations) would provide valuable insight into electrical signal transduction and practical sensor design. By integrating these investigations, future studies can establish a comprehensive computational framework that supports both diagnostic sensing and environmental remediation applications using doped coronene.

## Conclusion

This computational study utilized a combination of Density Functional Theory (DFT), Time-Dependent DFT (TD-DFT), and Quantum Theory of Atoms in Molecules (QTAIM) to investigate the interactions between formaldehyde and both pristine and metal-doped coronene. These methods were employed to model the binding and electronic properties of formaldehyde on coronene, and to explore the impact of aluminum and zinc doping on the material’s reactivity and stability. Specifically, the B97D functional was used in two phases (water and gas) with a consistent basis set of 6-311 + G(d) across all calculations. To ensure the reliability and accuracy of the results, the B97D method was validated by performing additional calculations using the WB97XD functional under the same conditions. The results obtained from both functionals showed excellent overlap, confirming the robustness of the computational approach and providing strong confidence in the accuracy of the predictions.

Aluminum (Al) and zinc (Zn) in coronene structure caused some different types of bond lengths, bond angles, and stability to the base structure of the materials. The base structure of coronene has consistent bond lengths for all carbon-carbon (C-C) bonds (1.430 Å) and all bond angles (approximately 120°). For aluminum-doped coronene (Al.Coronene), the Al-C1 bond length is 1.799 Å, the Al-C2 bond length is 1.819 Å, and the bond angles range from 112.68° to 123.65°. However, the bond length and angles of zinc-doped coronene (Zn.Coronene) were far greater than for Al.Coronene. Bond lengths of Zn-C1, Zn-C2, and Zn-C3 were 2.17 Å, 1.99 Å, and 1.98 Å, respectively. At the same time, the bond angles ranged from 79.42° to 101.23° for Zn.Coronene. The aforementioned changes in bond lengths and angles influence the cohesive energy (E_Coh_) of all types of coronene structures. The cohesive energy (E_Coh_) for the pristine coronene structure was − 148.71 kcal/mol. However, the cohesive energy of Al.Coronene was − 141.18 kcal/mol, and Zn.Coronene was − 145.99 kcal/mol. It can be inferred that Al.Coronene and Zn.Coronene have different stability compared to the original coronene structure due to the addition of aluminum and zinc.

This investigation also assessed the binding of formaldehyde to coronene derivatives. When comparing the Eads values for formaldehyde interacting with aluminum (Al) doped coronene to those for zinc (Zn) doped coronene and non-doped coronene, we found that Al-Coronene had the lowest Eads value, -39.57 kcal/mol (in water), indicating the strongest formaldehyde binding to this type of coronene compared to (Zn.Coronene@FA) -6.16 kcal/mol, and Coronen@FA: -4.01 kcal/mol. The reactivity values (HLG, chemical hardness, and softness) for Al.Coronene@FA supports the notion that aluminum doping greatly increases the reactivity of coronene and that the lower the HLG value, the greater the reactivity of the molecule is due to the increased number of reactive sites. The HLG for Al.Coronene@FA is 0.39 eV, the HLG for Zn.Coronene@FA = 1.19 eV and Coronene@FA = 1.79 eV.

Basic criteria such as recovery time, electrical conductivity, and adsorption energy were studied to evaluate the sensing performance of each of the complexes. All complexes showed the ability to adsorb formaldehyde; however, Al-doped coronene has the longest recovery time (1.08 × 10^13^ s) indicating that it is able to maintain a stable interaction with formaldehyde, making it an ideal candidate for long-term sensor applications, while Zn-doped coronene showed a much faster recovery time (3.34 × 10^–12^ s), making it much more suitable for applications where rapid reversibility is required. The recovery time of pristine coronene was significantly shorter (8.76 × 10^–14^ s). In addition to the above metrics, all complexes exhibited an increase in Electrical conductivity from formaldehyde adsorption, with Al-doped coronene exhibiting a maximum value of (2.85 × 10^9^ A.m^-2^), followed by Zn-doped coronene (2.43 × 10^9^ A.m^-2^), followed lastly by pristine coronene (2.15 × 10^9^ A.m^-2^).

The UV adsorption spectra revealed significant shifts in adsorption peaks due to formaldehyde adsorption, particularly for aluminum-doped coronene. In the water phase, pristine coronene showed a maximum adsorption wavelength (λmax) of 338 nm, with an exciton energy (Eex) of 3.66 eV. Zinc-doped coronene shifted to 398 nm (Eex = 3.10 eV), and aluminum-doped coronene exhibited a dramatic red shift to 579 nm (Eex = 2.13 eV), indicating its higher sensitivity to formaldehyde. In the gas phase, the trends were similar, with aluminum-doped coronene showing the largest shift to 613 nm (Eex = 2.02 eV), further confirming its sensitivity.

The Quantum Theory of Atoms in Molecules (QTAIM) and Non-Covalent Interaction (NCI) analyses provided complementary insights into the nature of the interactions between formaldehyde and the doped coronene structures. The QTAIM analysis revealed that aluminum-doped coronene exhibited strong Lewis acid-base interactions, suggesting a stable and efficient adsorption mechanism with formaldehyde, while zinc-doped coronene showed weaker interactions. The NCI analysis further confirmed these findings, highlighting the strength of the electron density interactions and the attractive non-covalent interactions in the aluminum-doped system, indicating its superior performance for sensing. Together, these methods reinforced that aluminum-doped coronene has the most favorable interaction with formaldehyde, making it the most promising candidate for high-sensitivity detection applications.

Finally, this study demonstrates that metal-doped coronene, especially those doped with aluminum, show exceptional promise as efficient sensors for formaldehyde. These materials exhibit enhanced reactivity, stability, and conductivity, making them ideal candidates for applications in environmental monitoring and biomedical diagnostics. Aluminum-doped coronene is particularly well-suited for long-term, stable sensing, while zinc-doped coronene is more suitable for applications requiring rapid reversibility. Further experimental work will be essential to validate these computational predictions and optimize these materials for real-world sensor applications.

## Supplementary Information

Below is the link to the electronic supplementary material.

Supplementary Material 1

## Data Availability

All data generated or analyzed during this study are included in this published article.
