# Supplementary material for: Computational study of metal doped coronene quantum dots for formaldehyde sensing and adsorption in medical and environmental applications
Source: Sci Rep. 2025 Dec 13;16:2761. doi: 10.1038/s41598-025-32667-7 (PMC12823697; doi:10.1038/s41598-025-32667-7)
Supplement: Supplementary file 1 — Supplementary Material 1 [file 41598_2025_32667_MOESM1_ESM.docx]

The geometric optimization convergence plots, also known as energy minimization profiles or stepwise energy optimization versus energy plots, are shown for each of the designed structures in Figures S1, S2, and S3. They show how the total electronic energy of each system evolves during the iterative geometric optimization process.

The graphs show the evolution of the total electronic energy as a function of the geometry-optimisation step for each system (Coronene, Al-Coronene, Zn-Coronene, and their corresponding Fa-bound complexes). In each structur, the optimisation starts from an initial trial geometry with relatively high energy; as the structure is iteratively relaxed, the energy decreases along the blue line and eventually reaches a plateau, where further optimisation steps no longer change the energy within numerical accuracy. This type of plot is essentially the “relaxation trajectory” on the potential-energy surface and is a standard way to monitor whether an optimisation is converging to a stationary point.

With this in mind, the behaviour seen in all structures is fully consistent with convergence to genuine local minima, even for the strongly distorted Zn systems. In every case the energy decreases smoothly and monotonically from the starting geometry, without oscillations or re-ascents that would indicate that the optimiser is having difficulty locating a stable structure. For the pristine Coronene and Al-Coronene molecules, the energy drops quickly and reaches a stable value after only a couple of steps, as expected for relatively rigid π systems with limited conformational freedom. For the Fa-bound and Zn-containing complexes, the energy decrease extends over more optimisation steps, reflecting the greater structural flexibility and the larger rearrangements needed to accommodate metal coordination and surface binding; however, here too the curves ultimately flatten into a clear plateau.

Crucially for the Zn-Coronene and Zn-Coronene@Fa structures, where the Zn-C coordination angles relaxed to ~84°, the optimisation traces show no subsequent increase in energy once the distorted geometry is reached. Instead, the energy approaches its final value asymptotically and then remains constant over the remaining iterations. This behaviour indicates that the optimiser is not sitting on, or sliding off, a saddle point, but has located a stationary point where the energy gradient is effectively zero. In other words, within the resolution provided by these optimisation profiles, the highly distorted Zn geometries correspond to well-defined local minima on the potential-energy surface, rather than artefacts of an incomplete or unstable optimisation.

| 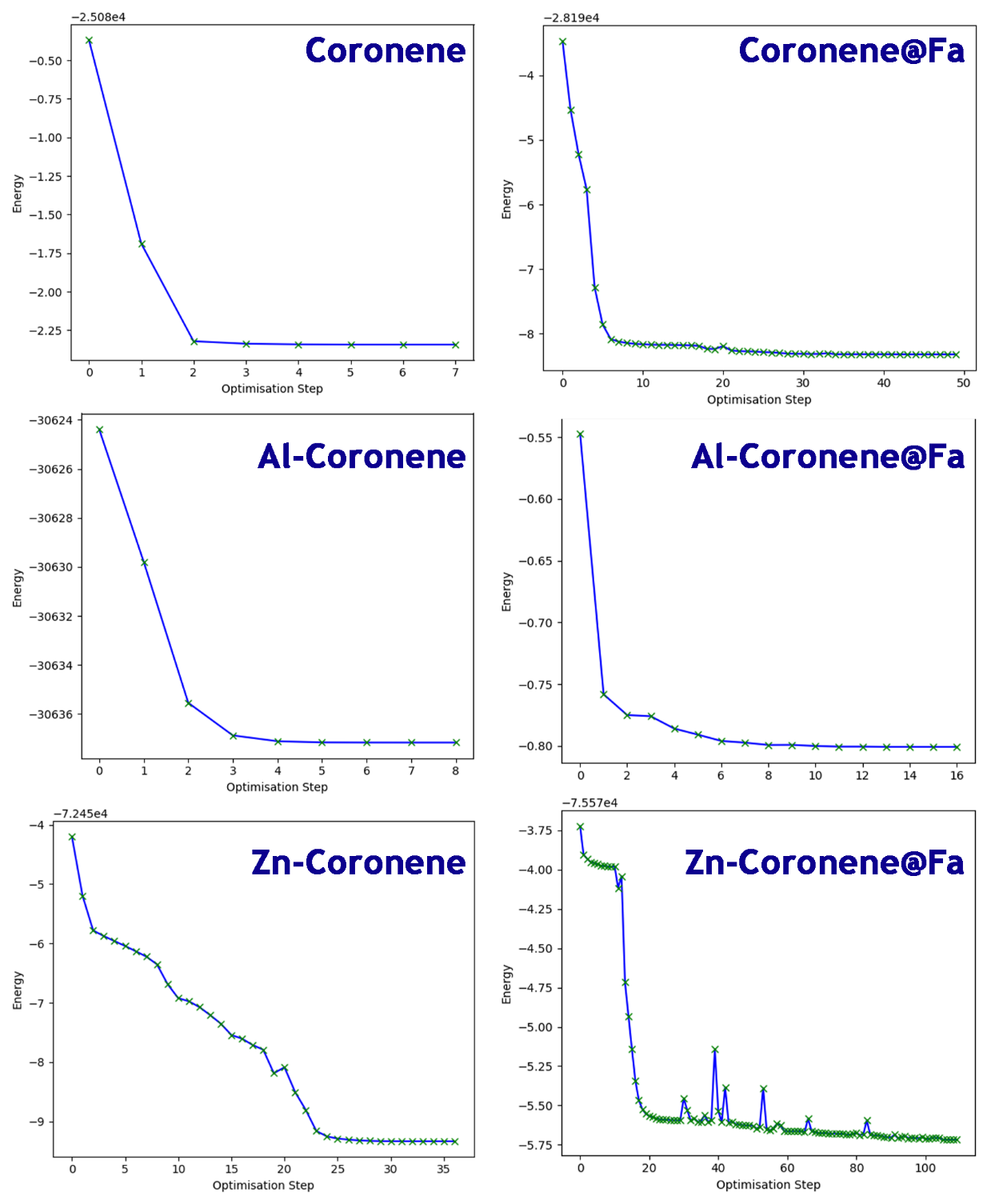 |
| --- |
| Fig S1. Geometric optimization convergence graphs for structures designed in the water phase using the computational method WB97XD/6-311+G(d). |

| 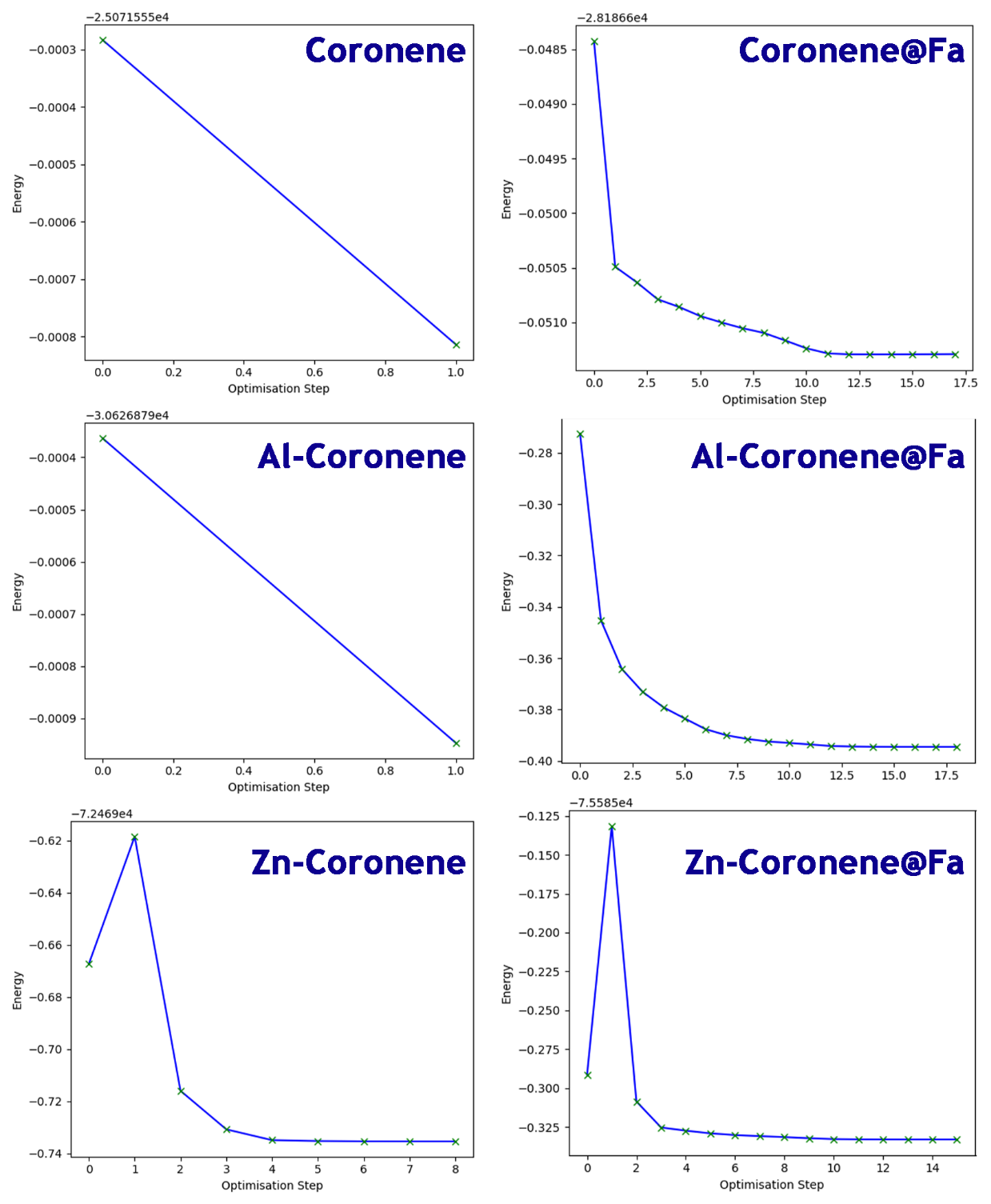 |
| --- |
| Fig S2. Geometric optimization convergence graphs for structures designed in the gas phase using the computational method B97D/6-311+G(d). |

| 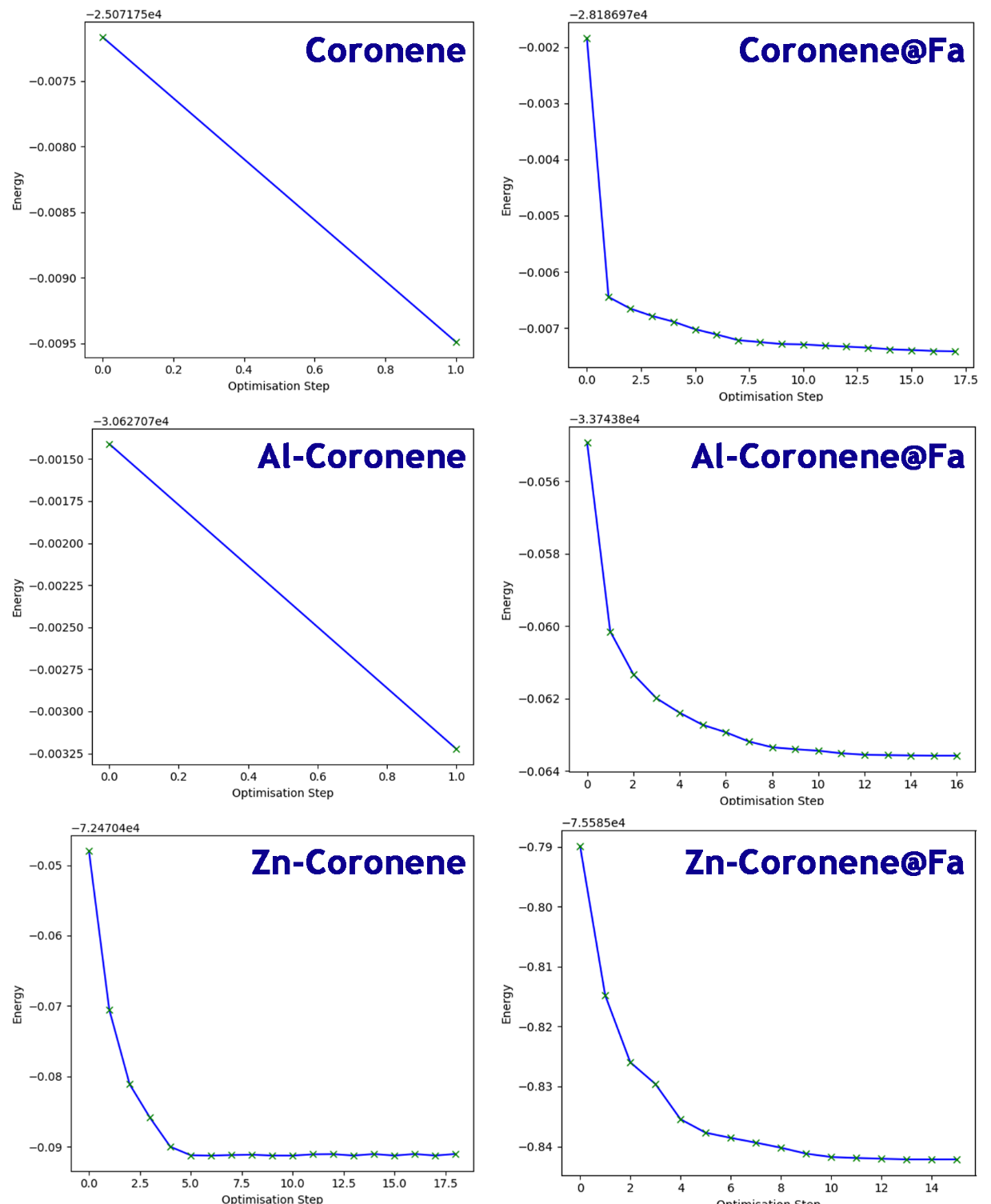 |
| --- |
| Fig S3. Geometric optimization convergence graphs for structures designed in the water phase using the computational method B97D/6-311+G(d). |

For the sake of repeatability of calculations, the complete coordinates of the designed structures were reported in Table S1.

| Table S1. X, Y, Z coordinates for each of the structures designed in the water phase and optimized using the computational surface b97d/6-311+g(d) |
| --- |
| Al.Coronene |
| %nprocshared=4  %chk=C:\Users\Windows 10\Desktop\Al-Cor.chk  # opt b97d/6-311+g(d) pop=nbo scrf=(cpcm,solvent=water) geom=connectivity  Title Card Required  0 2  C -3.40095231 -2.86145391 -0.00013900  C -1.97569031 -2.86145391 -0.00013900  C -1.29311231 -1.62920291 -0.00013900  C -2.02953931 -0.40442191 0.00003700  C -3.43798731 -0.42943391 -0.00007600  C -4.10636731 -1.68825891 -0.00013600  C 0.13577969 -1.60382891 -0.00009100  C -1.33706231 0.84572009 0.00040500  C 0.82825569 -0.35367391 0.00050900  C 0.77438469 2.10336109 0.00154000  C 0.01838569 3.31160009 0.00169300  C -1.35034131 3.28729109 0.00115200  C -2.06295531 2.05296709 0.00061600  C -3.48731831 2.00238409 0.00030600  C -4.15063231 0.80487109 0.00000000  H -5.25091331 0.77244509 -0.00015100  H -4.04345531 2.95232109 0.00037500  H -3.92301931 -3.83053491 -0.00014500  H -5.20710331 -1.69491291 -0.00013400  H 0.56299369 4.26819609 0.00220500  H -1.92857831 4.22393909 0.00117100  C -1.21970931 -4.06970091 -0.00032200  C 0.14903669 -4.04538991 -0.00066200  C 0.86165969 -2.81106691 -0.00051700  H -1.76429031 -5.02631691 -0.00023700  H 0.72726169 -4.98204791 -0.00090100  C 2.28602569 -2.76048091 -0.00048400  C 2.94934069 -1.56296891 0.00036000  C 2.23669269 -0.32867091 0.00091700  H 2.84215769 -3.71041191 -0.00098100  H 4.04962069 -1.53053891 0.00061000  C 2.19964669 2.10336609 0.00233300  H 2.72171069 3.07246709 0.00334000  C 2.90506969 0.93016609 0.00192500  H 4.00580969 0.93684909 0.00237200  Al 0.09183469 0.87110509 0.00079900 |
| Al.Coronene@FA |
| %nprocshared=4  %chk=C:\Users\Windows 10\Desktop\AlCor@FA-Water.chk  # opt b97d/6-311+g(d) scrf=(cpcm,solvent=water) pop=nbo geom=connectivity  out=wfn  Title Card Required  0 2  C 3.70201200 -1.29633800 -0.11363200  C 3.00731200 -0.04729100 0.04637300  C 1.58261500 -0.01654400 0.15674400  C 0.87086300 -1.31116100 0.02846900  C 1.59978500 -2.49437900 -0.36065600  C 3.02874900 -2.47362800 -0.34990200  C 0.92989100 1.31435700 0.09524200  C -0.54137400 -1.42115600 0.12634500  C -0.47685500 1.48534600 0.17364300  C -3.10040900 0.11880700 -0.33972600  C -3.48622400 -1.14422500 -0.87971800  C -2.68372100 -2.31197700 -0.84387200  C -1.26209000 -2.47382300 -0.49372900  C -0.52168900 -3.62125900 -0.92740700  C 0.86114300 -3.64304500 -0.78688500  H 1.42408700 -4.53320800 -1.08006300  H -1.03872900 -4.47482600 -1.37574900  H 4.79446000 -1.27353100 -0.14008400  H 3.57684400 -3.39355600 -0.57010800  H -4.39983900 -1.23653700 -1.48892400  H -3.11990400 -3.18486200 -1.34299300  C 3.76053900 1.17437800 -0.04151000  C 3.14304000 2.39328200 -0.20730400  C 1.71681200 2.48283600 -0.21774800  H 4.85075700 1.10208600 -0.06984000  H 3.73423600 3.29792700 -0.37246300  C 1.03888700 3.69030700 -0.57641100  C -0.34216000 3.74744300 -0.72291700  C -1.14148200 2.61246300 -0.36986300  H 1.64747800 4.56821300 -0.80888400  H -0.81345700 4.65588900 -1.10956000  C -3.43016400 1.42511100 -0.81032100  H -4.34372400 1.59273700 -1.40296900  C -2.56951700 2.54601900 -0.72359700  H -2.96288200 3.46737100 -1.16785100  Al -1.54916200 0.04534600 0.80360000  C -0.67216000 -0.61449000 3.36562900  H 0.09231100 -1.20454500 2.83673400  H -0.64362700 -0.51372900 4.45982700  O -1.59271500 -0.02314200 2.72783100 |
| Coronene |
| %chk=C:\Users\Windows 10\Desktop\Cor.chk  %nprocshared=4  # opt b97d/6-311+g(d) scrf=(cpcm,solvent=water) pop=nbo geom=connectivity out=wfn  Title Card Required  0 1  C 3.47291287 -1.90563386 0.02954199  C 3.04983733 -0.55702867 0.00356151  C 1.70919067 -0.28000823 -0.13657986  C 0.71740644 -1.41391965 -0.26363452  C 1.18969973 -2.71417482 -0.26454514  C 2.57630161 -2.94570321 -0.10476280  C 1.23923029 1.15637867 -0.17484004  C -0.63938972 -1.13701991 -0.37992523  C -0.11656269 1.43821971 -0.29096005  C -2.57366933 0.60748251 -0.65707955  C -3.47366091 -0.45683957 -0.87618601  C -3.04042147 -1.80685139 -0.82966504  C -1.55096946 -2.15927794 -0.59679915  C -1.09799409 -3.49224486 -0.60352220  C 0.27585818 -3.77140676 -0.42555050  H 0.62052362 -4.78433367 -0.41634081  H -1.79324943 -4.29329102 -0.74439465  H 4.51283005 -2.12484081 0.15372158  H 2.93872758 -3.95232617 -0.08871196  H -4.50093886 -0.24172530 -1.08433469  H -3.75283052 -2.59359767 -0.96532499  C 3.96441678 0.51532046 0.11317718  C 3.54564706 1.82890993 0.06018276  C 2.18066379 2.16692309 -0.09592091  H 5.00581113 0.30338944 0.23760812  H 4.27129735 2.61138665 0.13804051  C 1.75307086 3.50475994 -0.17418534  C 0.38151945 3.79525492 -0.35176552  C -0.55494398 2.74675054 -0.42731366  H 2.46491272 4.30039920 -0.10249625  H 0.05516592 4.81127355 -0.42986498  C -2.98496396 1.95028875 -0.79302846  H -4.01199447 2.16662180 -1.00113504  C -2.06075287 3.01860954 -0.66296287  H -2.40854382 4.02782813 -0.73657875  C -1.13508146 0.30156321 -0.25599152 |
| Coronene@FA |
| %chk=C:\Users\Windows 10\Desktop\Cor@FA.chk  %nprocshared=4  # opt b97d/6-311+g(d) scrf=(cpcm,solvent=water) pop=nbo geom=connectivity out=wfn  Title Card Required  0 1  C 3.46574744 -1.90139688 0.01799742  C 3.04097748 -0.55576867 0.01919099  C 1.69580325 -0.27950007 -0.07254161  C 0.70032501 -1.39571615 -0.18175862  C 1.17849711 -2.69506299 -0.25916145  C 2.56664500 -2.93420472 -0.12894724  C 1.21531441 1.14088929 -0.09417826  C -0.66574696 -1.11232851 -0.21047574  C -0.15272545 1.41458397 -0.12323005  C -2.54887007 0.59812597 -0.53188233  C -3.42545935 -0.44637795 -0.88668648  C -3.01832988 -1.78793557 -0.84549909  C -1.55203436 -2.13016935 -0.55588754  C -1.09363324 -3.45749493 -0.64397399  C 0.27291323 -3.74469947 -0.46727830  H 0.61921437 -4.75660965 -0.49911078  H -1.78409614 -4.24900564 -0.84811064  H 4.50678469 -2.12329620 0.12709432  H 2.92699486 -3.94152775 -0.14761023  H -4.42068517 -0.21078413 -1.20120379  H -3.72457311 -2.56920699 -1.03453740  C 3.95632886 0.51498588 0.10142689  C 3.53261187 1.82371226 0.03532745  C 2.16284188 2.15337525 -0.09176137  H 5.00000632 0.30609105 0.21097280  H 4.25709390 2.60994007 0.07858967  C 1.74053391 3.48413790 -0.21769114  C 0.37256045 3.76431374 -0.39462950  C -0.56780171 2.71771663 -0.38850652  H 2.45404217 4.28092434 -0.18707547  H 0.04776083 4.77389539 -0.53658056  C -2.94450288 1.92259665 -0.80489384  H -3.94856347 2.11467437 -1.12091361  C -2.04755251 2.99367579 -0.68040631  H -2.39107603 3.99899799 -0.80775962  C -1.75670356 -0.76855862 2.16692173  H -1.85218600 -1.67596884 1.60800332  H -1.93426129 -0.76894157 3.22208670  O -1.43558550 0.29907351 1.58329909  C -1.19829033 0.29958563 0.17312508  C:/Users/Windows 10/Desktop/Cor@FA.wfn |
| Zn.Coronene |
| %nprocshared=4  %chk=C:\Users\SasAn\Desktop\ZnCor.chk  # opt b97d/6-31g* scrf=(cpcm,solvent=water) geom=connectivity  Title Card Required  0 1  C 4.02190807 0.67501873 -0.03900494  C 2.79166766 1.38024028 -0.11948963  C 1.61497956 0.67837387 -0.35029161  C 1.71624926 -0.80364818 -0.44824915  C 2.91743224 -1.46126371 -0.33764143  C 4.08639312 -0.69753674 -0.16070393  C 0.29868026 1.59532588 -0.49377577  C 0.61024553 -1.60373607 -0.61401477  C -0.91774348 1.05448181 -0.75857524  C -2.49065480 -1.65381856 -0.74993985  C -2.28000393 -3.00521721 -0.33875210  C -0.86848862 -3.61023056 -0.27603581  C 0.49681058 -2.95373577 -0.45383969  C 1.73299415 -3.63218298 -0.42339290  C 2.92143025 -2.89616976 -0.39049847  H 3.85688403 -3.41561699 -0.39139472  H 1.76101256 -4.70178575 -0.41533696  H 4.92586475 1.22493672 0.12021982  H 5.03602689 -1.18824930 -0.11277810  H -3.12396783 -3.61920022 -0.10285001  H -0.83862250 -4.66005785 -0.07141253  C 2.75531899 2.80140724 0.02891298  C 1.59658245 3.55509973 -0.11840475  C 0.35976238 2.95599047 -0.43707484  H 3.66712221 3.31151956 0.25978159  H 1.64590697 4.61696599 0.00369000  C -0.83819934 3.69541388 -0.73790144  C -2.06622680 3.02239491 -1.11535416  C -2.10154183 1.61197380 -1.09752753  H -0.81989867 4.76419926 -0.69033153  H -2.93289525 3.58544057 -1.39241979  C -3.56280374 -0.82136216 -1.22276130  H -4.50748751 -1.27295108 -1.44307931  C -3.41187782 0.64721468 -1.40156115  H -4.27987180 1.15547585 -1.76646802  Zn -0.94830716 -0.73271541 -0.71575602 |
| Zn.Coronene@FA |
| %nprocshared=4  %chk=C:\Users\SasAn\Desktop\ZnCor@FA.chk  # opt b97d/6-311+g(d) scrf=(cpcm,solvent=water) geom=connectivity out=wfn  Title Card Required  0 1  C 4.06907800 0.70435000 0.03292200  C 2.82563000 1.41799200 -0.03063700  C 1.59975700 0.68980700 -0.00230600  C 1.65102200 -0.74541600 0.15619900  C 2.89427800 -1.43425500 -0.01411300  C 4.10558900 -0.67575000 -0.01161400  C 0.35030400 1.38010700 -0.33501400  C 0.45941400 -1.49329500 0.33957400  C -0.87340700 0.70211700 -0.56245600  C -2.52260100 -1.53646800 -0.31680600  C -2.11206100 -2.78829200 -0.82761800  C -0.85177800 -3.40450100 -0.64412300  C 0.38480500 -2.81585000 -0.19871400  C 1.63791800 -3.49693600 -0.43343200  C 2.84560100 -2.85014800 -0.25221700  H 3.78635600 -3.38710400 -0.39636800  H 1.61659100 -4.53573100 -0.77513400  H 4.99710300 1.28118800 0.03045400  H 5.06167000 -1.20192700 -0.07418000  H -2.77749600 -3.31478200 -1.53070300  H -0.74067800 -4.38913400 -1.11100600  C 2.80055700 2.84170800 -0.20582200  C 1.62269900 3.51467300 -0.44601200  C 0.38169200 2.80982200 -0.55674300  H 3.74888200 3.38395600 -0.17676400  H 1.62436800 4.59728700 -0.59748300  C -0.82937500 3.46734200 -0.92251100  C -2.01602200 2.77199200 -1.10768400  C -2.07122100 1.34702200 -0.93596200  H -0.80375800 4.54922400 -1.07688200  H -2.92320600 3.31235600 -1.39334000  C -3.53975700 -0.71520700 -0.91546300  H -4.48384000 -1.15115400 -1.27380200  C -3.33258000 0.62896500 -1.15587200  H -4.14210900 1.20860000 -1.60925500  Zn -1.22580600 -0.71054200 0.87721400  C -1.55629800 1.73921600 2.47080700  H -1.35802100 2.29480900 1.53786700  H -1.73953500 2.30187000 3.40531100  O -1.57895000 0.50160300 2.49732300  C:\Users\SasAn\Desktop\ZnCor@FA.wfn |
|  |
|  |
